# Bioactive, PDMS-containing shape memory composite scaffolds with accelerated degradation rates

**DOI:** 10.1016/j.polymer.2025.128653

**Published:** 2025-06-09

**Authors:** Brandon M. Nitschke, MaryGrace N. Wahby, Kaylee M. Breining, Melissa A. Grunlan

**Affiliations:** a Department of Biomedical Engineering, Texas A&M University, College Station, TX, 77843, United States; b Department of Materials Science and Engineering, Texas A&M University, College Station, TX, 77843, United States; c Department of Chemistry, Texas A&M University, College Station, TX, 77843, United States

**Keywords:** Bioactivity, Composite, Regenerative engineering, Scaffold, Polysiloxane, Polydimethylsiloxane, Semi-interpenetrating network, Bioglass

## Abstract

A self-fitting scaffold could enable a regenerative engineering approach to treat irregular craniomaxillofacial (CMF) bone defects. We have previously reported conformally fitting, shape memory polymer (SMP) scaffolds based on poly(ε-caprolactone) (PCL). The fitting temperature (i.e., melt temperature, *T*_*m*_) was tuned based on PCL architecture: *linear*-PCL-diacrylate (*linear*-PCL-DA; *T*_*m*_ = ~55 °C) or *star*-PCL-tetraacrylate (*star*-PCL-TA, *T*_*m*_ = ~45 °C). Scaffolds were also formed as semi-interpenetrating networks (semi-IPNs) by incorporating thermoplastic poly(L-lactic acid) (PLLA). The inclusion of a polydimethylsiloxane-dimethacrylate (PDMS-DMA) macromer and 45S5 Bioglass^®^ (BG) were independently shown to promote hydroxyapatite (HAp) mineralization, as well as to accelerate degradation. In this study, PDMS-containing, composite SMP scaffolds were prepared with varying macromer compositions and BG concentrations. PCL/PDMS co-matrix scaffolds were formed with either *linear*-PCL-DA or *star*-PCL-TA, and PDMS-DMA (75:25 wt%). PCL/PLLA/PDMS (75:12.5:12.5 wt%) co-matrix-semi-IPNs were also formed. BG was included at relatively low concentrations (5 and 10 wt%). Composite scaffolds were fabricated to concentrate BG on the pore walls via a modified solvent-cast particulate leaching (SCPL) approach with a fused salt/BG template. PDMS-containing scaffolds preserved shape memory behavior and were non-brittle. *In vitro* degradation rates were accelerated for PDMS-containing composite scaffolds, owing to a combination of phase separation of polymer components and hydrophilicity imparted by the BG. Additionally, robust bioactivity was observed for PDMS-containing composite scaffolds with HAp mineralization commencing in just 1 day (1X simulated body fluid; SBF).

## 1. Introduction

Owing to their irregular geometries, craniomaxillofacial (CMF) bone defects are challenging to treat. Autografting is the “gold standard” for treatment, but achieving a conformal fit with high tissue-to-graft contact within the defect is difficult and leads to premature graft resorption [1–5]. An off-the-shelf scaffold that could “self-fit” into irregular defects and support regeneration is a potentially superior alternative [6,7]. The scaffold should also be bioactive (i.e., promote the formation of carbonated hydroxyapatite [HAp, Ca_10_(PO_4_)_6_(OH)_2_]) [8,9]. HAp mineralization of a scaffold is essential to osteogenesis, as it promotes both osseointegration (i.e., bone-to-scaffold bonding) and osteoinductivity (i.e., mesenchymal stem cell [MSC] differentiation into osteoblasts) [10,11]. Bioceramics are commonly used to achieve bioactivity, and are frequently combined with biodegradable polymers to enhance degradation rates and reduce brittleness [12–14]. In particular, because of its robust bioactivity, 45S5 Bioglass^®^ (BG) [SiO_2_–Na_2_O–CaO–P_2_O_5_] is frequently used to prepare composite scaffolds [15–18].

Previously, we have fabricated self-fitting, shape memory polymer (SMP) (i.e., capable of thermally driven shape actuation) scaffolds based on poly(ε-caprolactone) (PCL) [19,20]. Others have since developed different SMP scaffold systems to achieve favorable osteogenic outcomes [21–24]. Our scaffolds were fabricated with UV-curable PCL macromers using a solvent-cast particulate leaching (SCPL) technique with a fused salt template for high porosity (~70%) and interconnected macropores (*d* ~ 220 μm). The PCL crystalline lamellae act as switching segments and crosslinks serve as netpoints, enabling the shape memory behavior of the scaffold. When exposed to warm saline above the PCL melt transition temperature (*T*_*m*_), the crystalline lamellae melt and the scaffold becomes malleable, allowing it to be press-fit into an irregular defect. Once implanted, continued exposure to warm saline triggers shape recovery (*R*_*r*_) to drive the scaffold outward and achieve a conformal fit. Finally, shape fixity (*R*_*f*_) locks the scaffold into shape once cooled below its *T*_*m*_. The *T*_*m*_ of SMP scaffolds was adjusted by altering the architecture of the PCL macromers [25–27]. To reduce the *T*_*m*_ of SMP scaffold, the architecture was changed from a *linear*-PCL-diacrylate (*linear*-PCL-DA, *T*_*m*_ = ~55 °C) to *star*-PCL-tetraacrylate (*star*-PCL-TA, *T*_*m*_ = ~45 °C). Thus, if an increased working time was needed, scaffolds based on *star*-PCL-TA would allow for prolonged irrigation at the surgical site with reduced risk of tissue necrosis [28]. SMP scaffolds were also formed as semi-interpenetrating networks (semi-IPNs) (i.e., comprised of a crosslinked network and an uncrosslinked thermoplastic component) via the incorporation of thermoplastic poly(L-lactic acid) (PLLA), resulting in higher modulus values [25]. The PCL/PLLA semi-IPN scaffolds (72:25 wt% PCL:PLLA) also exhibited faster degradation rates versus corresponding PCL-only scaffolds, owing to phase separation effects, which is expected to facilitate neotissue infiltration.

To bestow bioactivity to SMP scaffolds, we have independently evaluated two strategies: the introduction of a bioactive polysiloxane macromer [29], and the incorporation of BG to form composites [27]. PCL-DA was combined with polydimethylsiloxane-dimethacrylate (PDMS-DMA) to form co-matrix SMP scaffolds [29]. The hydrolysis of ester crosslinks formed by the PDMS-DMA, as well as the siloxane bonds (to form dimethylsilanediol) [30], allow for the observed degradation of PCL/PDMS scaffolds. PCL/PDMS scaffolds (75:25 wt% PCL:PDMS) exhibited HAp mineralization in simulated body fluid (SBF, 1X) at 4 weeks, and osteogenesis of cultured cells [31]. Compared to PCL-only scaffolds, faster rates of degradation were observed for the PCL/PDMS scaffolds due to phase separation of macromers. However, prior to mineralization, PCL/PDMS scaffolds exhibited reduced moduli stemming from the low glass transition temperature (*T*_*g*_) of PDMS (~−127 °C). In the second approach, BG-based composite SMP scaffolds were fabricated with matrices based on *linear*-PCL-DA and *star*-PCL-TA (*M*_*n*_ ~10 kg mol^−1^), as well as with semi-IPNs when combined with *linear*-PLLA or *star*-PLLA (*M*_*n*_ ~15 kg mol^−1^) (75:25 wt% PCL:PLLA). These composite scaffolds were fabricated via a modified SCPL method termed the “glass-in-salt mold” technique, where BG and salt were fused together in a template. Subsequently, the macromer solutions were UV-cured around the template, and the salt was extracted. The resulting composite scaffolds had BG concentrated on the pore walls rather than pore struts. This allowed for the use of BG at low levels (5 and 10 %), avoiding brittleness, but maximizing bioactivity of the surfaces that are first exposed to the physiological environment. These composite scaffolds began to mineralize in just 1 day in 1X SBF. Additionally, degradation rates were faster for composite scaffolds versus the corresponding BG-free scaffold. Overall, our previously reported SMP scaffolds have demonstrated high potential for bone regeneration in terms of non-cytotoxicity and *in vivo* regeneration [19,31–34].

**Fig. 1.**
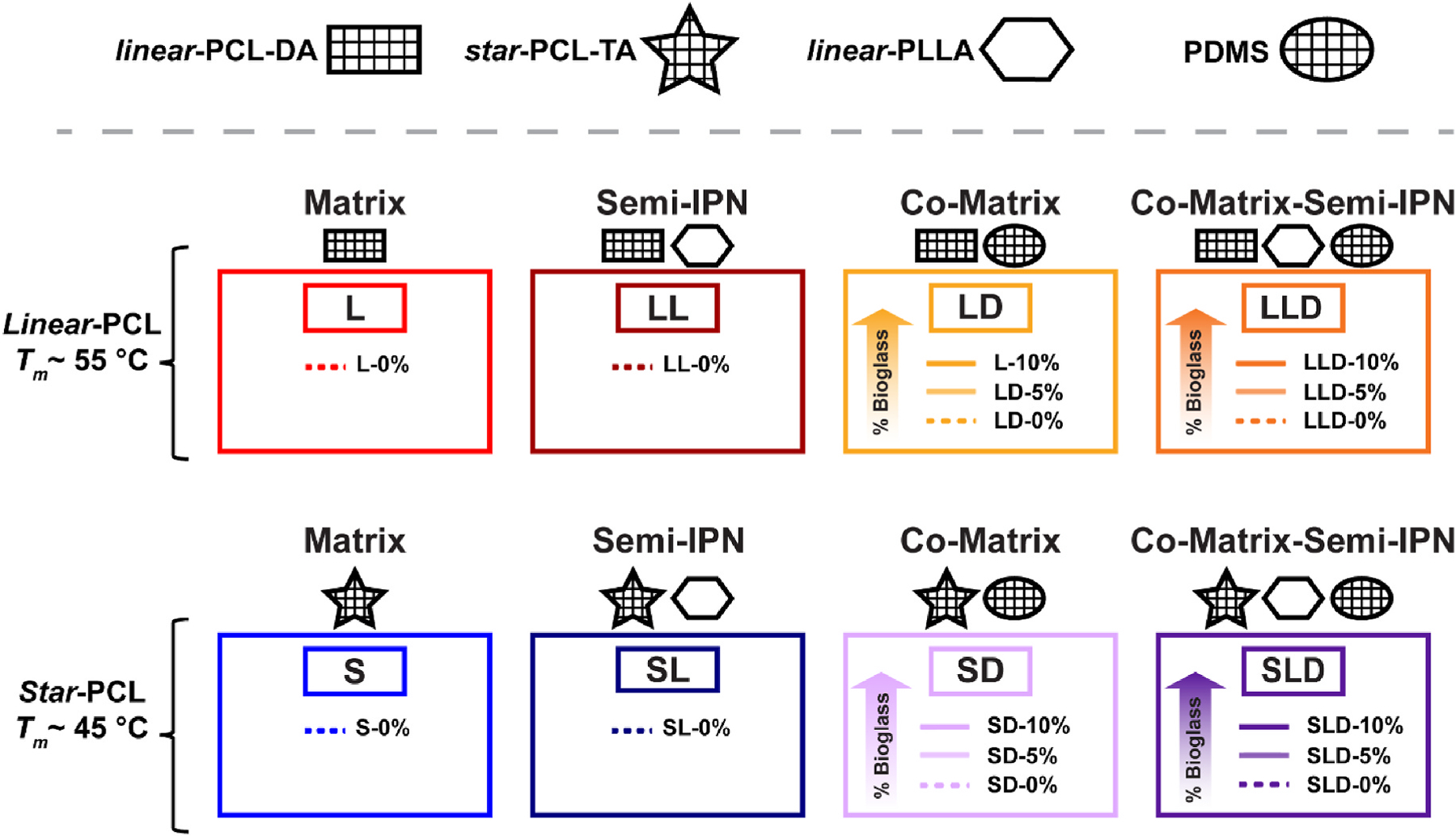
Scaffold designation based on macromer composition and % BG. *Control scaffolds* (i.e., no PDMS-DMA nor BG): *linear*-PCL-DA and *star*-PCL-TA each formed as matrix scaffolds; each combined with PLLA (75:25 wt%) to form semi-IPN scaffolds. *PDMS-containing scaffolds: linear*-PCL-DA and *star*-PCL-TA each combined with PDMS-DMA (75:25 wt%) to form co-matrix scaffolds; PCL, PLLA, and PDMS (75:12.5:12.5 wt%) combined to form co-matrix-semi-IPN scaffolds. Percentages signify BG incorporation into scaffolds with respect to the total macromer wt.

Herein, toward creating potently bioactive SMP scaffolds with favorably faster rates of degradation, composites were formed by the inclusion of bioactive PDMS-DMA in the scaffold bulk as well as with BG concentrated on the scaffold pore walls. These PDMS/BG composite scaffolds were fabricated via our previously established “glass-in-salt mold” SCPL approach. The macromer compositions (i.e., combinations of PCL and PDMS macromers, or with PCL, PLLA, and PDMS macromers) and BG levels were systematically varied (Fig. 1, Table S1). PCL and PDMS co-matrix scaffolds were formed with *linear*-PCL-DA/PDMS-DMA **(LD)** and *star*-PCL-TA/PDMS-DMA **(SD)** (PCL:PDMS = 75:25 wt%). PCL, PLLA, and PDMS co-matrix-semi-IPN scaffolds were formed with *linear*-PLLA to create *linear*-PCL-DA/PLLA/PDMS-DMA **(LLD)** and *star*-PCL-TA/PLLA/PDMS-DMA **(SLD)** (PCL:PLLA:PDMS = 75:12.5:12.5 wt%). BG levels in scaffolds were varied (0, 5, and 10 wt%) based on the total macromer weight. The resulting composite SMP scaffolds were evaluated for their morphological, mechanical, thermal, shape memory, degradation, and bioactive properties.

## 2. Materials and methods

### 2.1. Materials

*Linear*-PCL-diol (*linear*-PCL-diol, *M*_*n*_ = 10 kg mol^−1^ per manufacturer), 4-di-methylaminopyridine (DMAP), triethylamine (Et_3_N), acryloyl chloride, potassium carbonate (K_2_CO_3_), anhydrous magnesium sulfate (MgSO_4_), sodium chloride (NaCl), (3S)-*cis*-3,6-dimethyl-1,4-dioxane-2,5-dione (L-lactide), ε-caprolactone, ethylene glycol, pentaerythritol, tin(II) 2-ethyl-hexanoate (Sn(Oct)_2_), octamethylcyclotetrasiloxane (D_4_), triflic acid, hexamethyldisilazane (HMDS), *N*-vinyl-2-pyrrolidone (NVP), 2,2-dimethoxy-2-phenylacetophenone (DMP), deuterated chloroform (CDCl_3_), sodium hydroxide (NaOH), sodium bicarbonate (NaHCO_3_), potassium chloride (KCl), potassium phosphate dibasic trihydrate (K_2_HPO_2_⋅3H_2_O), magnesium chloride hexahydrate (MgCl_2_⋅6H_2_O), hydrochloric acid (HCl), calcium chloride (CaCl_2_), sodium sulfate (Na_2_SO_4_), tris(hydroxymethyl)aminomethane (tris), Alizarin Red S, acetic acid, and solvents were purchased from Sigma-Aldrich. 1,3-bis(3-methacryloxy-propyl)tetramethyldisiloxane was purchased from Gelest. All solvents and ethylene glycol were dried over 4 Å molecular sieves prior to use. Bioglass^®^ (45S5, 10 μm) was purchased from Mo-Sci Corp (Rolla, MO, USA).

### 2.2. Syntheses

Glassware and Teflon-coated stir bars used for reactions were rinsed with acetone and dried at 120 °C prior to synthesis. After polymer synthesis and purification, structures were confirmed with ^1^H NMR spectroscopy (400 MHz spectrometer operating in Fourier Transform mode with CDCl_3_ as the standard).

*Star-*PCL-tetrol (*M*_*n*_ = 10 kg mol^−1^) was synthesized via ring opening polymerization (ROP) per a prior protocol [25]. Briefly, ε-caprolactone (20.0 g), pentaerythritol (0.278 g, 88:1, [M]:[I]), and Sn(Oct)_2_ were added into a round-bottom (rb) flask and allowed to react overnight (ON) at 120 °C. The crude product was dissolved in CH_2_Cl_2_, precipitated in cold CH_3_OH, and vacuum filtered and dried (room temperature [RT], ON, 30 in. Hg) to yield purified *star*-PCL-tetrol. The structure and *M*_*n*_ were confirmed via ^1^H NMR and agreed with the previous report [25].

*Linear-*PCL-diol (*M*_*n*_ = 10 kg mol^−1^) and *star-*PCL-tetrol (*M*_*n*_ = 10 kg mol^−1^) were acrylated to produce UV-curable *linear*-PCL-DA and *star*-PCL-TA macromers, respectively, per prior reports [20,25]. *Linear*-PCL-diol or *star*-PCL-tetrol (20.0 g) were dissolved in DCM (0.17 g mL^−1^) and combined with DMAP (6.6 mg for *linear*-PCL-diol; 13.2 mg for *star*-PCL-tetrol). The rb flask was purged for ~3 min with N_2_, and Et_3_N (0.56 mL for *linear*-PCL-diol; 1.12 mL for *star*-PCL-tetrol) and acryloyl chloride (0.65 mL for *linear*-PCL-diol; 1.30 mL for *star*-PCL-tetrol) were each subsequently added dropwise to the flask. The reaction proceeded under positive N_2_ pressure for 30 min, followed by under reflux for 16 h at 55 °C. The crude products were purified per the prior protocol [25], to yield *linear*-PCL-DA and *star*-PCL-TA. Acrylation (>90 %) was confirmed by ^1^H NMR and agreed with the prior report [25].

PDMS-dimethacrylate (PDMS-DMA, *M*_*n*_ = 6 kg mol^−1^) was synthesized via a ROP by combining D_4_ (55.607 g, 187 mmol) and 1,3-bis(3-methacryloxypropyl)tetramethyldisiloxane (4.393 g, 11.4 mmol) in a rb flask in the presence of triflic acid (80 μL, 0.904 mmol). The reaction was allowed to continue ON at RT. HMDS (189 μL, 0.904 mmol) was added to quench the reaction and was allowed to stir for 1 h at RT. Finally, the solution was filtered (Ashless, Grade 42) and dried under vacuum (RT, ON, 30 in. Hg) to purify the product. PDMS-DMA structure and *M*_*n*_ were confirmed with ^1^H NMR, and agreed with the prior report [29].

*Linear*-PLLA (*M*_*n*_ = 15 kg mol^−1^) was synthesized according to a prior report [25]. Briefly, L-lactide (6.0 g), ethylene glycol (25 mg), and Sn (Oct)_2_ were combined in a rb flask and allowed to react ON at 120 °C. After removal from heat, the crude product was dissolved in CHCl_3_, precipitated in cold CH_3_OH, filtered, and dried under vacuum (RT, ON, 30 in. Hg). The structure and *M*_*n*_ of the purified product were confirmed with ^1^H NMR, and agreed with the previous report [25].

### 2.3. Scaffold fabrication

#### “Glass-in-salt mold SCPL.”

Scaffolds were fabricated according to an established procedure to form composites with BG concentrated on the pore walls [27]. Briefly, sieved salt (10 g, 459 ± 70 μm) and the designated BG wt% (0, 5, or 10 wt% w.r.t. total macromer wt) was added into a 20 mL scintillation vial and thoroughly mixed with a spatula. DI water (~0.81 mL) was added in four portions to the NaCl and BG mixture, with mechanical stirring after each addition for even distribution of BG throughout the template. The NaCl/BG mixture was then centrifuged (3220 rpm, 15 min) to fuse the template. After centrifugation, the fused template was dried to remove water (RT, ON, 30 in. Hg). Macromer solutions were prepared by dissolving the polymer(s) in DCM (0.17 g mL^−1^). A photoinitiator solution (10 wt% DMP in NVP) was added to the macromer solution at 15 vol% and mixed atop a shaker plate (3 min, 150 rpm). Then, ~5 mL of the macromer solution was added to each NaCl/BG template and the vials were capped and centrifuged (1260 rpm, 10 min) to diffuse the macromer solution throughout the template. Vials were uncapped and placed on a UV plate for 7 min under foil-covered beakers to cross-link the macromer(s) (UV-Transilluminator, 6 mW cm^−2^, 365 nm). The vials were air dried in a fume hood ON and then dried under vacuum for 5 h (RT, 30 in. Hg). Next, NaCl was removed by soaking vials in a water and ethanol solution (1:1 by vol.) for ~5 days with daily solution changes. The wet scaffolds were air dried (48 h), subsequently vacuum dried (RT, ON, 30 in. Hg), and finally annealed under vacuum (30 in. Hg) at 85 °C (1 h) or 180 °C (5 min for semi-IPN-containing scaffolds). After resting for 48 h, resulting cylindrical specimens (*d* ~ 12 mm) were sliced into discs (*t* ~ 2 mm) (Vibratome, Leica VT 1000 S) and biopsy punched (Integra Miltex, 6 mm) to produce *d* ~ 6 mm × *t* ~ 2 mm final scaffold specimens.

### 2.4. Film fabrication

Films were prepared with the aforementioned macromer solutions for porosity calculations (with and without BG) and evaluation of phase separation (without BG). Macromer solutions with BG were stirred at 500 rpm ON in a rb flask. The photoinitiator solution (15 vol%) was added to the macromer solution, vortexed (1 min), and the solution transferred into a silicone mold (*d* ~ 50 mm × *t* ~2 mm, McMaster-Carr) between glass slides. Then, the mold was UV-cured (UV-Trans-illuminator, 6 mW cm^−2^, 365 nm) for 3 min per side. The films were air dried ON, vacuum dried (30 in. Hg) for 5 h, and then soaked in EtOH atop a shaker plate (150 rpm, 3 h). After removal from EtOH and air-drying ON, films were annealed under vacuum (30 in. Hg) at 85 °C (1 h) or 180 °C (5 min for semi-IPN-containing films). Films were biopsy punched (Integra Miltex, 6 mm) to yield final specimens (*d* ~ 6 mm × *t* ~2 mm).

### 2.5. Film characterization

#### 2.5.1. Confocal microscopy imaging and surface roughness

Film surfaces were assessed with a scanning confocal laser microscope (Evident LEXT^™^ OLS5100-LAF). Area roughness (arithmetical mean height*, Sa*) was calculated on ~1000 μm^2^ areas (*N* = 3) in accordance with ISO 25178. OLS51-BSW software was used for calculations.

### 2.6. Scaffold characterization

#### 2.6.1. Thermal gravimetric analysis (TGA)

TGA (TA Instruments Q50) testing of scaffolds (*N* = 3, *d* ~ 4 mm × *t* ~ 2 mm) was carried out in platinum pans in a N_2_ environment. Samples were heated from RT to 600 °C (heating rate = 10 °C min^−1^).

#### 2.6.2. Sol content

Scaffolds (*N* = 3) were each submerged in 10 mL DCM in scintillation vials, and placed on a shaker plate (150 rpm, 48 h). Scaffolds were then removed, briefly rinsed with DCM, and dried under vacuum (RT, ON, 30 in. Hg). Initial and final masses of scaffolds were used to calculate sol content (%).

#### 2.6.3. Pore size, porosity (%), and pore interconnectivity

Scaffold pore sizes were analyzed via scanning electron microscopy (SEM, Tescan Vega 3, 10 kV accelerating voltage) images (*N* = 2). Prior to imaging, scaffold cross-sections were coated with 10.0 nm Au–Pt. Pore size measurements (*N* = 10) were obtained from pores along the diagonal midline of images with ImageJ software. Scaffold porosity (*N* = 3) was calculated via equation (1), after determining the density of films and scaffolds compositions [35]. Pore interconnectivity of scaffolds (*N* = 3) was calculated using a water-wicking test per Frassica et al. [36] Briefly, specimens were submerged in 10 mL of DI water and placed atop a shaker plate (150 rpm, 24 h). Scaffolds were removed from the vials and weighed (*mass_total_*). After using a Kimwipe to wick away interconnected water, scaffolds were weighed once again (*mass_interconnected_*). Pore interconnectivity was calculated via equation (2).


(1) $$\text{Porosity}(\%)=\frac{\rho_{\text{solid}\;\text{film}}-\rho_{\text{porous}\;\text{scaffold}}}{\rho_{\text{solidfilm}}}*100$$

(2) $$\text{Pore}\;\text{Interconnectivity}(\%)=\frac{{\text{mass}}_{\text{total}}-{\text{mass}}_{\text{interconnected}}}{{\text{mass}}_{\text{total}}}*100$$

#### 2.6.4. Alizarin Red S staining

Alizarin Red S staining was used to confirm BG incorporation into scaffolds (*N* = 3) by staining for calcium. Briefly, Alizarin Red S was dissolved in DI water (2% w/v). The solution and scaffolds were combined in individual well plates and allowed to soak atop a shaker plate (5 min, 150 rpm). Scaffolds were removed from the wells and rinsed with DI water. To extract the stain from scaffolds for quantification, scaffolds were soaked in an aqueous solution of 10% acetic acid and 20% CH_3_OH in a new well plate placed atop a shaker plate (15 min, 150 rpm). Scaffolds were removed from the well plate, and the absorbances of each well were read on a spectrophotometer (Cytation 5 Biotek) at 450 nm.

#### 2.6.5. Compressive mechanical properties

Compressive mechanical properties of scaffold specimens were assessed (Instron 5944). Specimens (*N* = 5) were subjected to a preload force of 0.1 N. The strain was then zeroed, and a strain rate of 1.5 mm min^−1^ was applied until 85% strain. Compressive modulus (*E*), strength (*CS*), and toughness were all calculated utilizing a MATLAB code on the resulting stress-strain curves. *E* was calculated from the slope of the initial linear region (0–10% strain). *CS* was determined from the stress at 85% strain. Toughness was calculated by integrating the stress-strain curve up to 85% strain.

#### 2.6.6. Thermal properties

PCL and PLLA *T*_*m*_ and % crystallinity for scaffolds (*N* = 3, *d* ~ 4 mm × *t* ~ 2 mm, ) were determined via differential scanning calorimetry (DSC, TA Instruments Q100). Scaffolds were sealed in Al hermetic pans and heated and cooled at 5 °C min^−1^. Non-semi-IPN-containing scaffolds were heated to 100 °C and cooled to 0 °C, and semi-IPN-containing scaffolds were heated to 200 °C and cooled to 0 °C. The cycle was repeated twice to eliminate thermal history, and reported values were obtained from the second cycle. The *T*_*m*_ was determined from the endothermic melt peak’s maximum value, and % crystallinity (%*X*_*c*_) was calculated with equation (3). Δ*H*_*m*_ is the enthalpy of fusion from the endothermic melt peak and $\Delta H_{\text{c}}^{\circ }$ is the enthalpy of fusion of theoretical 100 % crystalline PCL (139.5 J g^−1^) [37] or PLLA (93.0 J g^−1^) [38]. *w* is the mass fraction of the PCL or PLLA (e.g., *w* = 0.75 for PCL and *w* = 0.125 for PLLA in co-matrix-semi-IPNs).


(3) $$\%X_{c}=\frac{\Delta H_{m}}{\Delta H_{c}^{\circ }*w}$$

#### 2.6.7. Shape memory behavior

##### 2.6.7.1. Qualitative assessment.

###### Irregular model defect.

Scaffold specimens (*N* = 3, *d* ~ 6 mm) were tested for their “self-fitting” properties into an irregular model defect. Briefly, the scaffolds were submerged in water 5 °C higher than their respective PCL *T*_*m*_ (i.e., 60 °C for scaffolds based on *linear*-PCL-DA, and 50 °C for scaffolds based on *star*-PCL-TA) for 1 min. Next, the scaffolds were press-fit into the model defect, cooled to RT (3 min), and removed from the defect. Finally, the scaffold was resubmerged in the warm water for 1 min and allowed to shape recover. The process was repeated for a second time to eliminate thermal history, and photographs were taken to document all four stages of the process.

##### 2.6.7.2. Quantitative assessment.

###### R_f_ and R_r_ of scaffolds in a circular defect.

Scaffold specimens (*N* = 3, *d* ~ 6 mm) were also evaluated in a quantitative manner per a prior report [25]. The scaffold initial diameter was measured, and scaffolds were subsequently submerged for 1 min in warm water per the qualitative test. Next, the scaffold was removed from the warm water and press-fit into a model circular defect (~5 mm). Scaffolds were cooled to RT (3 min), removed from the circular defect, and the new diameter was measured. Then, scaffolds were resubmerged in the water bath for 1 min and the scaffold diameter was measured in its “recovered shape.” The entire process was repeated a second time to eliminate thermal history. Reported values of *R*_*f*_ and *R*_*r*_ were from the second cycle using equations (4) and (5).


(4) $$R_{f}(N)=\frac{\varepsilon_{u}(N)}{\varepsilon_{m}}*100$$

(5) $$R_{r}(N)=\frac{\varepsilon_{i}(N)}{\varepsilon_{r}(N)}*100$$

*ε*_*u*_ *(N)* is the scaffold diameter after press-fitting into the mold, *ε*_*m*_*(N*) is the mold diameter, *ε*_*i*_ *(N)* is the scaffold diameter after shape-recovery from the mold, *ε*_*r*_ *(N)* is the initial scaffold diameter, and *N* is the cycle number.

#### 2.6.8. Accelerated degradation

Scaffold specimens (*N* = 3 per time-point) were exposed to base-catalyzed conditions (0.2 M NaOH) in compliance with ASTM F1635. Each specimen was immersed in 10 mL NaOH, sealed in a 20 mL scintillation vial, and placed inside an incubator (VWR Benchtop Shaking Incubator Model 1570, 37 °C, 60 rpm). At 24, 48, 72, 96, 120, 144, and 168 h, specimens were removed, thoroughly rinsed with DI water, and vacuum dried (RT, 48 h, 30 in. Hg). Results were reported as mass remaining (%) calculated from the initial and final masses of each scaffold. Each scaffold was used for only a single time-point.

#### 2.6.9. Bioactivity

SBF (1X) was prepared as described according to Kokubu [39]. Scaffold specimens (*N* = 3 per time-point) were each placed in a sealed centrifuge tube (50 mL) along with ~10 mL of SBF at 37 °C. Scaffolds were removed at 1-day, and 2-week time-points, rinsed with DI water, and dried under vacuum (RT, ON, 30 in. Hg). Prior to imaging, scaffolds were coated with Au–Pt (10.0 nm). Specimens were analyzed via SEM/energy-dispersive X-ray spectroscopy (SEM/EDS, Tescan Vega 3, accelerating voltage of 10 kV) to assess HAp mineralization on scaffolds.

### 2.7. Statistical analyses

Data was analyzed in GraphPad Prism via ANOVA tests, where a *p*-value of <0.05 was considered statistically significant. *N* = number of independent specimens tested. All data was reported as a mean ± standard deviation.

## 3. Results and discussion

### 3.1. Scaffold fabrication

PCL-based SMP composite scaffolds were prepared with a combination of PDMS macromer (PDMS-DMA) and BG (Fig. 1, Table S1). These were fabricated via a “glass-in-salt mold” SCPL technique that concentrates the BG on the pore wall surfaces [27]. PCL/PDMS co-matrix scaffolds were prepared based on the combination with *linear*-PCL-DA/PDMS-DMA **(LD)** and *star*-PCL-TA/PDMS-DMA **(SD)** (PCL: PDMS = 75:25 wt%). Co-matrix-semi-IPN scaffolds were formed with *linear*-PLLA to create *linear*-PCL-DA/PLLA/PDMS-DMA **(LLD)** and *star*-PCL-TA/PLLA/PDMS-DMA **(SLD)** (PCL:PLLA:PDMS = 75:12.5:12.5 wt%). All scaffold compositions maintained PCL at 75 wt% to ensure retention of shape memory behavior. PDMS-DMA comprised the remaining 25 wt% (for **LD** and **SD** co-matrix scaffolds), or 12.5 wt% (for **LLD** and **SLD** co-matrix-semi-IPNs scaffolds; with PLLA at 12.5 wt%). BG levels in scaffolds were varied (0, 5, and 10 wt%) based on the total macromer weight. In addition, “non-PDMS-containing” scaffolds without BG were formed as controls from *linear*-PCL-DA **(L)**, *star*-PCL-TA **(S)**, *linear*-PCL-DA/PLLA **(LL)**, (75:25 wt%), and *star*-PCL-TA/PLLA **(SL)** (75:25 wt%).

**Fig. 2.**
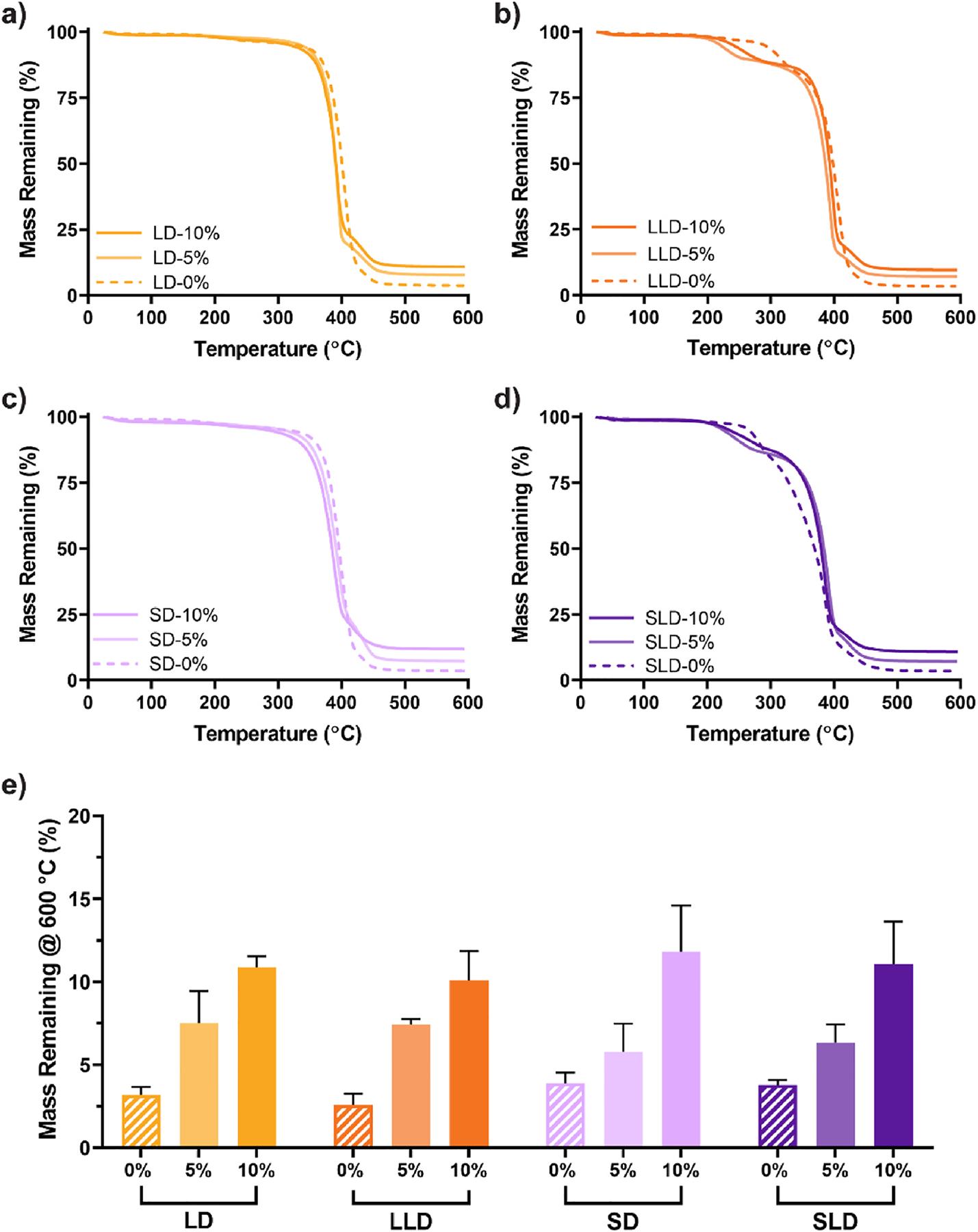
Representative TGA curves of PDMS-containing scaffolds: **(a)** LD, **(b)** LLD, **(c)** SD, and **(d)** SLD scaffold series. **(e)** TGA plateau values at 600 °C. % refers to wt % of BG.

Scaffold sol content values were assessed to verify adequate cross-linking of UV-curable macromers. PCL/PDMS co-matrix scaffolds (**LD** and **SD**) produced sol content values < 6%, indicating that PCL and PDMS macromers were well cross-linked, even in the presence of BG (Fig. S1, Table S2). PCL/PLLA/PDMS co-matrix-semi-IPN scaffolds (**LLD** and **SLD**) displayed the expectedly higher sol content values (~11–16%) stemming from uncrosslinked thermoplastic *linear*-PLLA incorporated at 12.5 wt%. Targeted BG levels were also verified by analyzing TGA plateaus at 600 °C, and indicated successful BG incorporation into the scaffolds (Fig. 2, Table S3). TGA analysis also confirmed PLLA incorporation ~12.5 wt% into **LLD** and **SLD** co-matrix-semi-IPN scaffolds based on mass loss around 300 °C. Alizarin Red S staining likewise verified the BG presence in scaffolds by staining for calcium (Fig. S2, Table S4). Absorbance values of scaffolds with 0% BG were low (~0.1), whereas scaffolds with 5% and 10% BG had higher values (~0.7–~1.4). Analysis of scaffold pore size and morphology revealed all compositions maintained a targeted pore size of ~240 μm (Figure S3, Figure S4, Table S5) in the optimal range for osseointegration and osteogenesis [40]. All scaffolds had high pore interconnectivity (45–60%) (Fig. S5, Table S5) and high porosity (~60–65%) (Fig. S6, Table S5) which are expected to also support osteogenesis [40–42].

### 3.2. Compressive mechanical properties

**Fig. 3.**
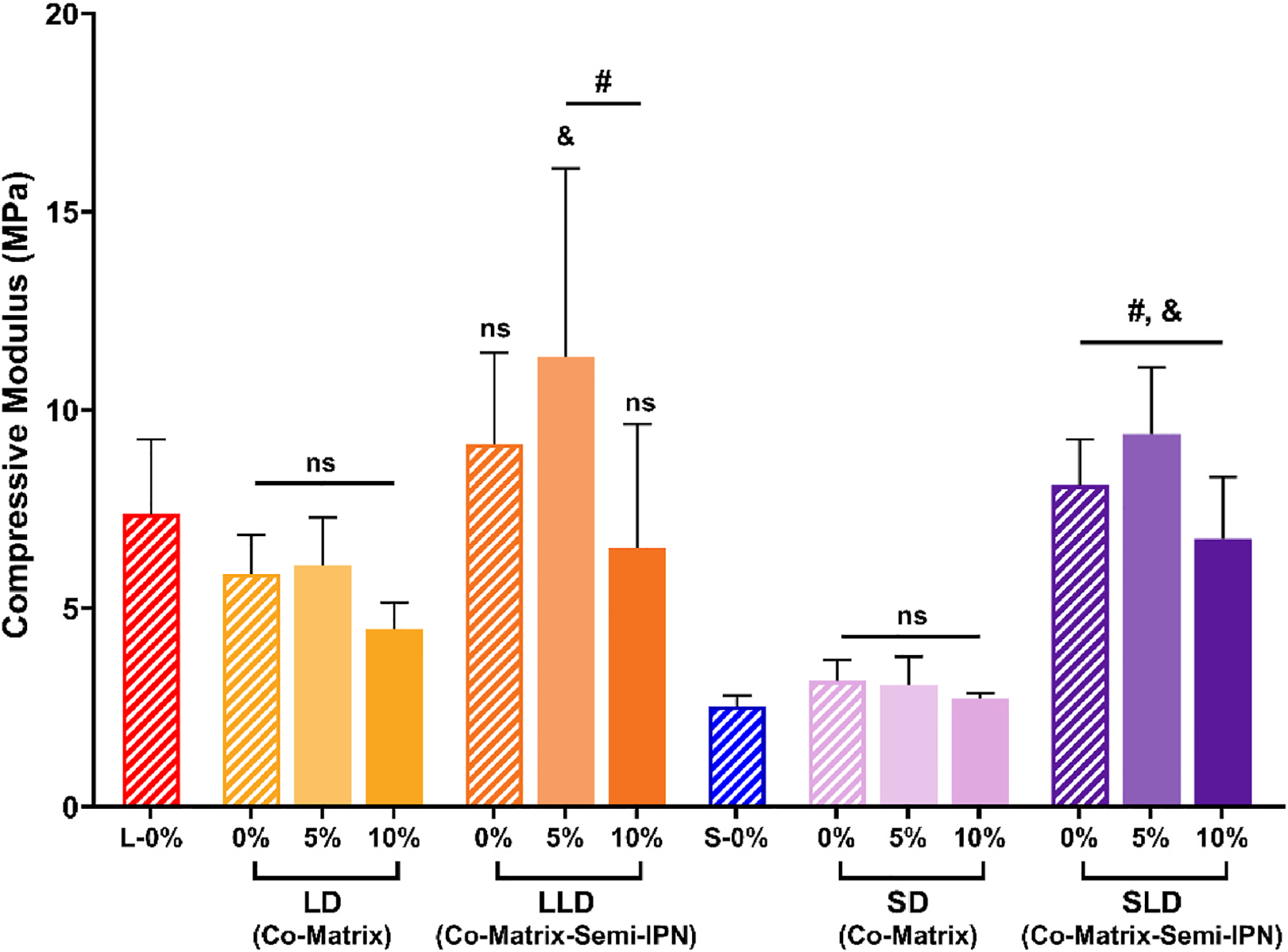
Compressive moduli (*E*) values of scaffolds; % refers to wt% of BG. ^&^*p* < 0.05 vs. analogous PCL-only scaffold; ^#^*p* < 0.05 vs. analogous co-matrix scaffold of the same BG wt%; *ns* = no significant difference vs. analogous PCL-only scaffolds. (Note: L-0% and S-0% data previously reported) [27].

The impact of macromer composition and BG levels on PDMS-containing scaffold compressive modulus (*E*) was determined via static compression testing (Fig. 3, Table S6). PCL macromer architecture played a primary role, with higher *E* values displayed for scaffolds based on *linear*-PCL-DA versus *star*-PCL-TA owing to the reduced crystallinity of the latter. For PCL/PDMS co-matrix scaffolds (**LD** and **SD**), a reduction in *E* versus the corresponding PCL-only scaffolds (**L** and **S**, respectively) was expected based on the low *T*_*g*_ (~−127 °C) of PDMS at 25 wt%. However, this reduction was mitigated, owing to an increase in crosslink density stemming from the lower molecular weight of PDMS-DMA (~6 kg mol^−1^) versus PCL macromers (~10 kg mol^−1^). Indeed, the *E* of **LD-0%** was similar to that of **L-0%**, and likewise the *E* of **SD-0 %** was similar to that of **S-0%**. With the inclusion of BG, **LD-5%** and **LD-10%**, as well as **SD-5 %** and S**D-10%** also did not produce a significant change in *E* values, attributed to the relatively low amount of BG and localization on the pore walls. The PCL/PLLA/PDMS semi-IPN-co-matrix scaffolds (having 12.5 wt% each of PDMS-DMA and PLLA), exhibited enhanced *E* values due to the high *T*_*g*_ (~60 °C) and *T*_*m*_ (~160 °C) of PLLA. All compositions (except **LLD-0%)** had statistically higher *E* values versus compared to the corresponding PCL/PDMS co-matrix compositions with the same BG wt%. Additionally, **LLD-5%**, **SLD-0%**, **SLD-5%**, and **SLD-10%** had significantly higher *E* values versus the corresponding PCL-only matrix scaffolds (**L** and **S** types). The incorporation of BG did not have a substantial impact on *E* values of PCL/PLLA/PDMS co-matrix-semi-IPN scaffolds, as also observed for PCL/PDMS co-matrix scaffolds. Compressive testing was also used to evaluate compressive strength (*CS*) and toughness values (Fig. S7, Table S6). Notably, **LD-5%** and **LLD-5%** with significantly higher *CS* and toughness values versus the **LD-0%** and **LLD-0%,** respectively. All scaffolds were non-brittle, and withstood 85% strain without fracture. The avoidance of scaffold brittleness is essential to resist post-surgical deformation and fracture during healing [43].

### 3.3. Thermal properties

The PCL *T*_*m*_ represents the temperature required for “self-fitting” the scaffold within the irregular defect. PCL crystallinity was also expected to impact mechanical properties and degradation rates. Thus, PCL *T*_*m*_ and % crystallinity of scaffolds were determined (Figure S8, Figure S9, Table S7). PCL/PDMS co-matrix scaffolds and PCL/PLLA/PDMS semi-IPN-co-matrix scaffolds with 0% BG all maintained similar PCL *T*_*m*_ and % crystallinity values when prepared with *linear*-PCL-DA (~55 °C; ~40%) or *star*-PCL-TA (~45 °C; ~30%). The relatively reduced *T*_*m*_ and crystallinity of *star*-PCL-TA may be credited to its higher crosslink density [26]. Some reports have shown that the incorporation of bioceramics into PCL scaffolds can significantly decrease crystallinity [44, 45]. Herein, the addition of 5 and 10 wt% BG did not produce substantial changes in scaffold PCL crystallinity, except for **LLD-10%**, which was less than the **L-0%** control scaffold. This favorable retention of PCL crystallinity of composite scaffolds is attributed to low BG levels and localization on pore walls. PLLA *T*_*m*_ and % crystallinity were also assessed for PCL/PLLA/PDMA co-matrix-semi-IPN scaffolds (Figure S8, Figure S9, Table S7). In the absence of BG, scaffolds exhibited PLLA crystallinity (a *T*_*m*_ at ~160 °C; ~35–45% crystallinity). However, BG-containing scaffolds exhibited a loss of PLLA crystallinity. This was attributed to the higher affinity of hydrophilic PLLA to BG, as compared to that of more hydrophobic PCL and BG [27]. However, as noted above, despite the loss of PLLA crystallinity for composite scaffolds, enhanced *E* values were observed.

### 3.4. Shape memory behavior

The self-fitting ability of the scaffolds, stemming from PCL crystallinity, was evaluated in terms of shape recovery (*R*_*r*_) and shape fixity (*R*_*f*_) via qualitative and quantitative shape memory tests. *R*_*r*_ represents the scaffold’s ability to expand and fill an irregular defect as it is driven to recover to its original shape. The scaffold’s ability to retain its new shape within the defect is represented by *R*_*f*_. During these tests, scaffolds were submerged in warm water for 1 min to ensure melting of PCL lamellae (60 °C for scaffolds based on *linear*-PCL-DA, and 50 °C for scaffolds based on *star*-PCL-TA), press-fit into a defect, cooled to RT, and removed from the defect to evaluate *R*_*f*_. The scaffold was subsequently removed from the defect and resubmerged in the designated warm water for 1 min to evaluate *R*_*r*_. During the qualitative test, each scaffold specimen (*d* ~ 6 mm) was press-fit into an irregular defect to mimic the complex geometries of CMF defects (Fig. S10). Visually, shape fixity and recovery were excellent for all PDMS-containing composite scaffolds compared to the corresponding scaffolds without BG. The quantitative test determined percentage values of *R*_*f*_ and *R*_*r*_ following press-fitting into a circular mold (*d* ~ 5 mm). All *R*_*f*_ and *R*_*r*_ values were near 100%, indicating outstanding shape memory properties (Table S8). The retention of shape memory behavior for composite PDMS-containing scaffolds is attributed to retention of PCL crystallinity.

### 3.5. Degradation

**Fig. 4.**
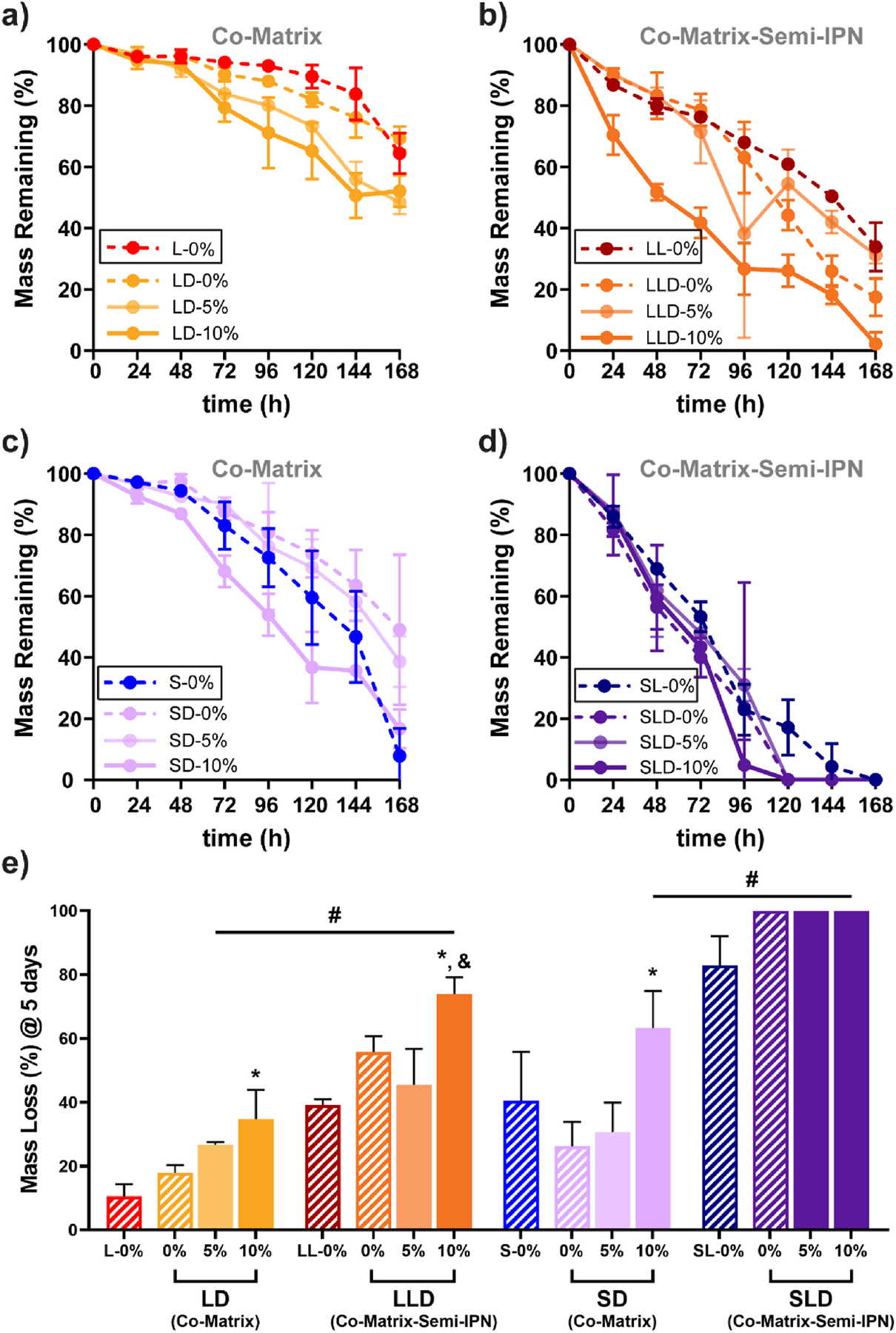
Scaffold mass loss over 7 days (0.2 M NaOH, 37 °C, 60 rpm) of **(a)** LD, **(b)** LLD, **(c)** SD, and **(d)** SLD. Non-PDMS-containing “controls” are designated with black boxes in the legend. **(e)** Mass loss for scaffolds at 5 days; ^#^*p* < 0.05 vs. analogous PCL-only scaffold; *p < 0.05 vs. 0 % BG of similar macromer composition; ^&^*p* < 0.05 vs. analogous 0 % BG semi-IPN compositions. % refers to wt% of BG. (Note: L-0% and S-0% data previously reported) [27].

The degradation time of PCL (~2 years), being far slower than the rate of neotissue formation (i.e., 3–6 months), limits its utility as a scaffold for bone regeneration [46,47]. Versus a PCL scaffold based only on *linear*-PCL-DA, we have demonstrated that degradation rates could be accelerated by changes to the macromer composition. For instance, scaffolds prepared with *star*-PCL-TA exhibited accelerated degradation due to reduced crystallinity [26]. Faster degrading scaffolds were also formed with the inclusion of PLLA (i.e., PCL/PLLA semi-IPNs) [25] as well as PDMS-DMA (i.e., PCL/PDMS co-matrices) [29]. Accelerated degradation rates were attributed to polymer phase separation effects, permitting enhanced water penetration. In another study, the inclusion of BG to scaffolds based on *linear*-PCL-DA or *star*-PCL-DA was shown to increase degradation rates due to greater hydrophilicity [27]. In particular, degradation was accelerated for composite PCL/PLLA semi-IPN scaffolds versus corresponding composite PCL-only scaffolds. In this present study, scaffolds that contained both PDMS-DMA and BG were expected to synergistically promote faster degradation rates, particularly if PLLA was also included. PCL/PDMS co-matrix and PCL/PLLA/PDMS co-matrix-semi-IPN scaffolds were subjected to *in vitro* degradation studies in basic conditions (Fig. 4, Fig. S11, Table S9). PDMS-containing scaffolds based on *star*-PCL-TA degraded faster than the corresponding scaffolds based on *linear*-PCL-DA due to lower PCL crystallinity (~30% versus ~40%). PCL/PLLA/PDMS co-matrix-semi-IPN scaffolds also degraded faster than the corresponding PCL/PDMS co-matrix scaffolds. Degradation rates of PDMS-containing scaffolds were increased by the inclusion of BG, and rates increased with BG concentration (5 to10 wt%). The fastest degradation was observed for the PCL/PLLA/PDMS co-matrix-semi-IPN prepared with *star*-PCL-TA and 10 wt% BG (**SLD-10%**). The accelerated degradation is attributed to the combination of enhanced hydrophilicity from the BG as well as phase separation of the PCL, PLLA, and PDMS macromers. Scanning confocal microscopy images revealed greater phase separation and surface roughness for PCL/PDMS co-matrix films (**LD** and **SD**) versus PCL-only films (**L** and **S**), as did PCL/PLLA/PDMS co-matrix-semi-IPN films (**LLD** and **SLD**) versus PCL/PLLA films (**LL** and **SL**) (Fig. S12, Table S10). The further increase in degradation rates with the inclusion of BG can be attributed to the hydrophilicity of the BG. Overall, PCL-based scaffolds containing both BG and PDMS, particularly in combination with PLLA, exhibited faster rates of degradation.

### 3.6. Bioactivity

**Fig. 5.**
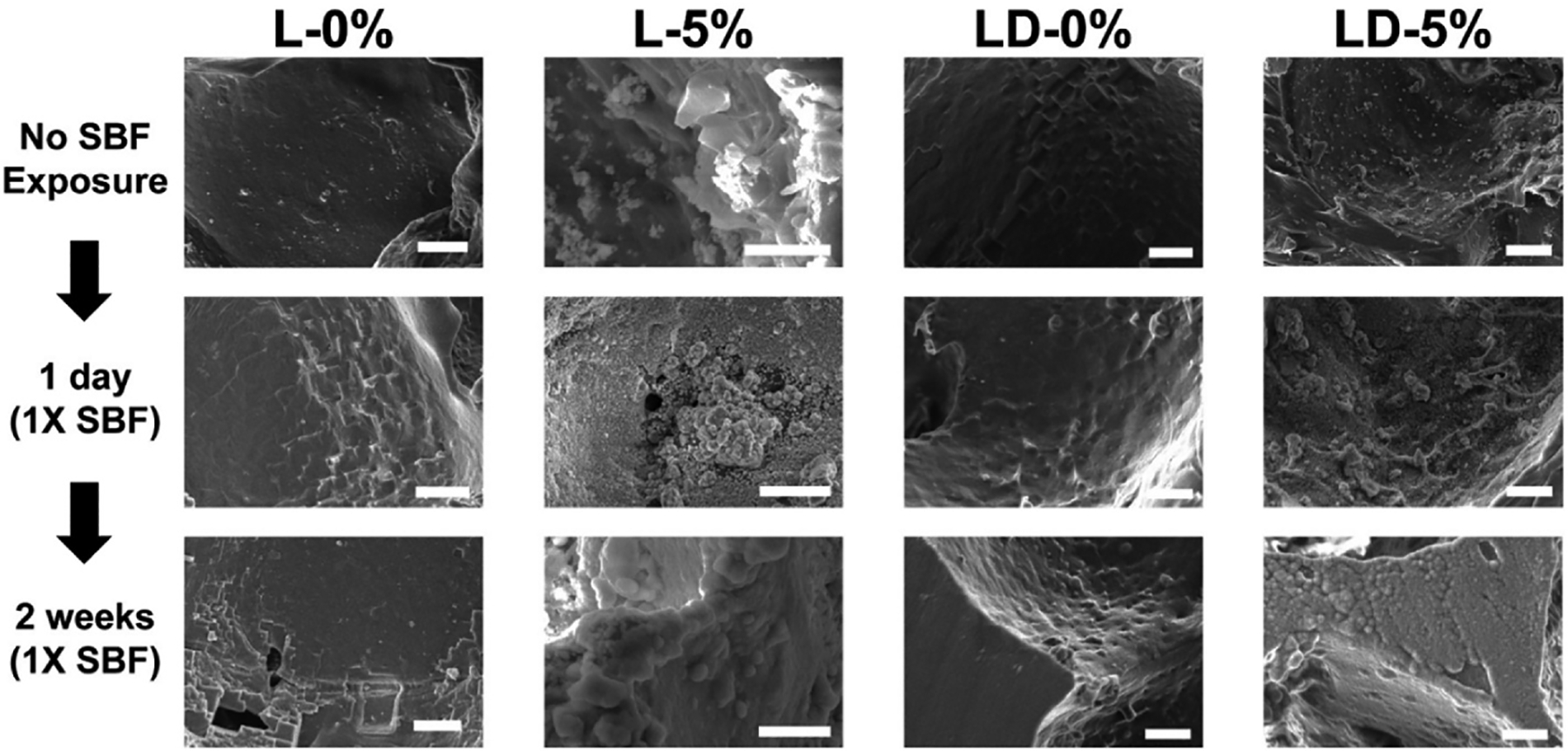
SEM images of scaffolds prior to and after 1X SBF exposure. (Scale bars = 20 μm). % refers to wt % of BG. (Note: L-0% and L-5% images previously reported) [27].

Scaffold bioactivity can be assessed *in vitro* via HAp mineralization following exposure to SBF [39]. In separate prior reports, we demonstrated that PCL/PDMS co-matrix scaffolds mineralized at 4 weeks (1X SBF, 37 °C) [29], while PCL-only and PCL/PLLA semi-IPN scaffolds with 5 and 10 wt% BG mineralized in just 1 day [27]. In this study, we likewise evaluated the bioactivity of scaffolds. SEM images of scaffolds prior to submersion in SBF were recorded (Fig. S13). After 1 day and 2 weeks in SBF, all BG-containing scaffolds mineralized, regardless of the inclusion of PDMS (Fig. 5, Fig. S14, Fig. S15). HAp has a Ca/P molar ratio of ~1.67 [48], such that SEM/EDS can be used for its identification. For the composite PCL/PDMS co-matrix scaffold (**LD-5%**), the CaP ratio increased from 1.49 (1 day) to 1.65 (2 weeks) (Fig. S16). While robust in HAp mineralization in just 1 day, the extent of HAp mineralization between BG-containing scaffolds with or without PDMS could not be visually discerned. Beyond acellular mineralization, cells associated with a scaffold can also give rise to HAp mineralization and osteogenic response. For instance, in a prior study, PCL/PDMS scaffolds (without BG) exhibited a pro-osteogenic effect after 2 weeks of cell culture compared to scaffolds without PDMS [31]. Thus, the presence of PDMS throughout the scaffold, in addition to the BG localized on pore wall surfaces are expected to synergistically promote bioactivity.

## 4. Conclusions

An “off-the-shelf”, self-fitting scaffold with robust bioactivity and accelerated degradation rates could enable a regenerative engineering strategy to treat complex CMF bone defects. We have previously reported on PCL-based SMP scaffolds capable of conformal fitting. Compared to scaffolds based on *linear*-PCL-DA, degradation rates were increased when prepared with *star*-PCL-TA, as well as when either were combined with thermoplastic PLLA to form semi-IPNs or with PDMS-DMA to form co-matrices. In separate studies, bioactivity was imparted by the inclusion of PDMS, and with the introduction BG, with more rapid *in vitro* HAp mineralization observed with BG. In this study, composite SMP scaffolds were formed with both PDMS-DMA and BG for a synergistic combination. PCL/PDMS co-matrix scaffolds were formed with *linear*-PCL-DA or *star*-PCL-TA and PDMS-DMA (75:25 wt%), and PCL/PLLA/PDMS (75:12.5:12.5 wt%) co-matrix-semi-IPNs formed by including PLLA. BG was included at 5 and 10 wt%, with BG localized on the pore walls by a modified SCPL protocol with a fused NaCl/BG template. The targeted level of BG in composite scaffolds was confirmed via TGA. Scaffolds displayed interconnected macropores (average pore size ~240 μm) and high porosity (~70%). For all composite scaffolds, shape memory behavior was retained owing to the retention of PCL crystallinity. If prolonged surgical site irrigation was necessary, scaffolds based on *star*-PCL-TA (*T*_*m*_ = ~45 °C) would permit fitting at a lower temperature and hence greater tissue safety versus those based on *linear*-PCL-DA (*T*_*m*_ = ~55 °C). Favorable to resisting post-surgical fracture, all scaffolds were non-brittle. This is attributed to the low levels of BG and the concentration of BG on pore walls rather than pore struts. Due to higher % crystallinity, *E* values of scaffolds were greater for those based on *linear*-PCL-DA (~40%) versus *star*-PCL-TA (~30%). Despite the low *T*_*g*_ (~−120 °C) of PDMS, PCL/PDMS co-matrix scaffolds had similar *E* values and may be due to higher crosslink density versus corresponding PCL-only scaffolds. PCL/PLLA/PDMS co-matrix-semi-IPN scaffolds had higher *E* values versus corresponding PCL/PDMS co-matrix scaffolds. Degradation rates (0.2 M NaOH, 37 °C) were accelerated by the combination of PDMS-DMA and BG in composite scaffolds, and is attributed to enhanced hydrophilicity from the BG and phase separation of the macromers. PCL/PLLA/PDMS co-matrix-semi-IPN scaffolds degraded faster than the corresponding PCL/PDMS co-matrix scaffolds, and rates increased with BG concentration (5 to 10 wt%). The PCL/PLLA/PDMS co-matrix-semi-IPN scaffold prepared with *star*-PCL-TA and 10 wt% BG (**SLD-10 %**) exhibited the fastest degradation. Greater phase separation was indeed observed via scanning confocal microscopy for PCL/PLLA/PDMS co-matrix-semi-IPNs. HAp mineralization of scaffolds following exposure to 1X SBF (37 °C) began at just 1 day and was more pronounced at 2 weeks. This favorable bioactivity for scaffolds with just 5 and 10 wt% BG is attributed to the concentration of BG at the pore walls, where interactions can be maximized with the local environment. The degree of HAp mineralization between BG-containing scaffolds with or without PDMS could not be distinguished. However, given the prior observation that “non-BG” PCL/PDMS co-matrix scaffolds produced an osteogenic response with cultured cells, future studies may reveal PDMS and BG provide enhanced cellular bioactivity. Overall, PDMS-containing composite scaffolds demonstrate great potential to heal irregular CMF bone defects by virtue of their bioactivity and enhanced degradation rates.

## Supplementary Material

1

## Data Availability

Data will be made available on the Texas Data Repository (TDR).

## Appendix A. Supplementary data

Supplementary data to this article can be found online at https://doi.org/10.1016/j.polymer.2025.128653.
