# Supplementary material for: Bioactive, PDMS-containing shape memory composite scaffolds with accelerated degradation rates
Source: Polymer (Guildf). Author manuscript; Available in PMC 2026 Jun 4. (PMC13232763; doi:10.1016/j.polymer.2025.128653)
Supplement: 1 [file NIHMS2177192-supplement-1.pdf]

Supporting Information

Bioactive, PDMS-containing shape memory composite scaffolds with accelerated degradation rates

Brandon M. Nitschke,[1] MaryGrace N. Wahby,[1] Kaylee M. Breining,[2]

*and Melissa A. Grunlan[1,2,3]**

[1]Department of Biomedical Engineering, Texas A&M University, College Station, Texas 77843, United States.

[2]Department of Materials Science and Engineering, Texas A&M University, College Station, Texas 77843, United States.

[3]Department of Chemistry, Texas A&M University, College Station, Texas 77843, United States.

*Corresponding author email: mgrunlan@tamu.edu

24 Pages

16 Figures

10 Tables

Table S1. Nomenclature of scaffold compositions.

	Notation	Bioglass	Macromers			
			Linear-PCL-DA or *Star*-PCL-TA		PDMS	*Linear*-PLLA
		wt% w.r.t. macromer	Architecture	Macromer wt%	Macromer wt%	Macromer wt%
Matrix	L-0%	0%	*Linear*	100%	-	-
Co-Matrix	LD-0%	0%	*Linear*	75%	25%	-
	LD-5%	5%		75%	25%	-
	LD-10%	10%		75%	25%	-
Semi-IPN	LL-0%	0%	*Linear*	75%	-	25%
Co-Matrix-Semi-IPN	LLD-0%	0%	*Linear*	75%	12.5%	12.5%
	LLD-5%	5%		75%	12.5%	12.5%
	LLD-10%	10%		75%	12.5%	12.5%
Matrix	S-0%	0%	*Star*	100%	-	-
Co-Matrix	SD-0%	0%	*Star*	75%	25%	-
	SD-5%	5%		75%	25%	-
	SD-10%	10%		75%	25%	-
Semi-IPN	SL-0%	0%	*Star*	75%	-	25%
Co-Matrix-Semi-IPN	SLD-0%	0%	*Star*	75%	12.5%	12.5%
	SLD-5%	5%		75%	12.5%	12.5%
	SLD-10%	10%		75%	12.5%	12.5%

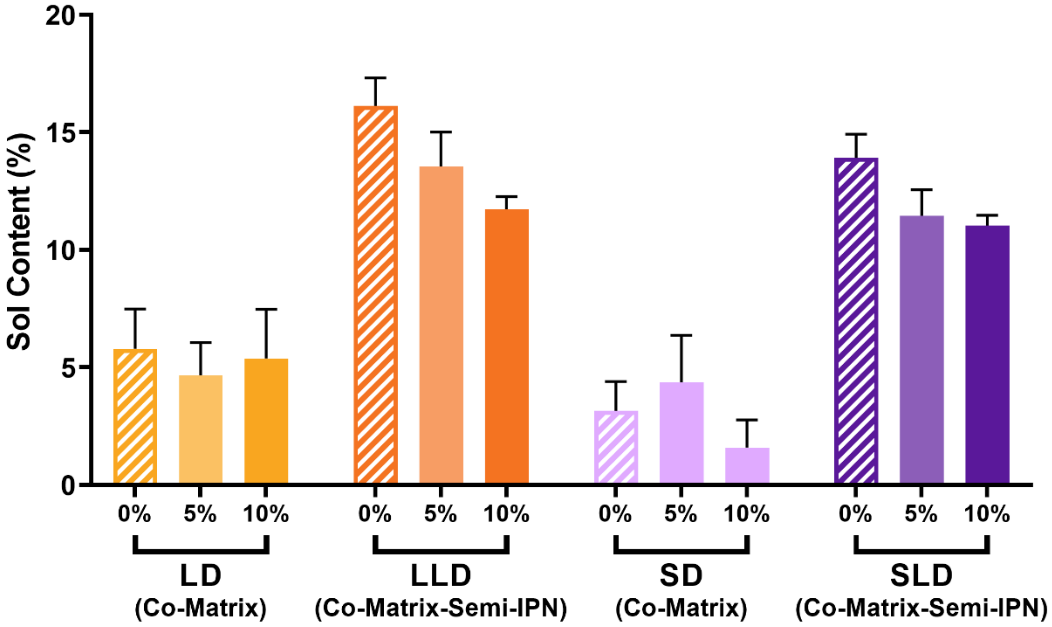

Figure S1. Sol content (%) of scaffolds. % refers to wt% of BG.

Table S2. Sol content (%) of scaffolds. % refers to wt% of BG.

Scaffold	Sol Content (%)
**LD-0%**	5.79 ± 1.69
**LD-5%**	4.66 ± 1.40
**LD-10%**	5.38 ± 2.09
**LLD-0%**	16.12 ± 1.20
**LLD-5%**	13.55 ± 1.46
**LLD-10%**	11.72 ± 0.54
**SD-0%**	3.15 ± 1.24
**SD-5%**	4.37 ±1.99
**SD-10%**	1.59 ± 1.18
**SLD-0%**	13.91 ± 1.00
**SLD-5%**	11.45 ± 1.11
**SLD-10%**	11.03 ± 0.44

Table S3. TGA wt% plateau values at 600 °C. % refers to wt% of BG.

Scaffold	Mass (%) remaining at 600 °C
**LD-0%**	3.21 ± 0.46
**LD-5%**	7.51 ± 1.95
**LD-10%**	10.88 ± 0.67
**LLD-0%**	2.59 ± 0.68
**LLD-5%**	7.44 ± 0.31
**LLD-10%**	10.11 ± 1.75
**SD-0%**	3.89 ± 0.63
**SD-5%**	5.79 ± 1.69
**SD-10%**	11.82 ± 2.79
**SLD-0%**	3.78 ± 0.30
**SLD-5%**	6.34 ± 1.09
**SLD-10%**	11.08 ± 2.56

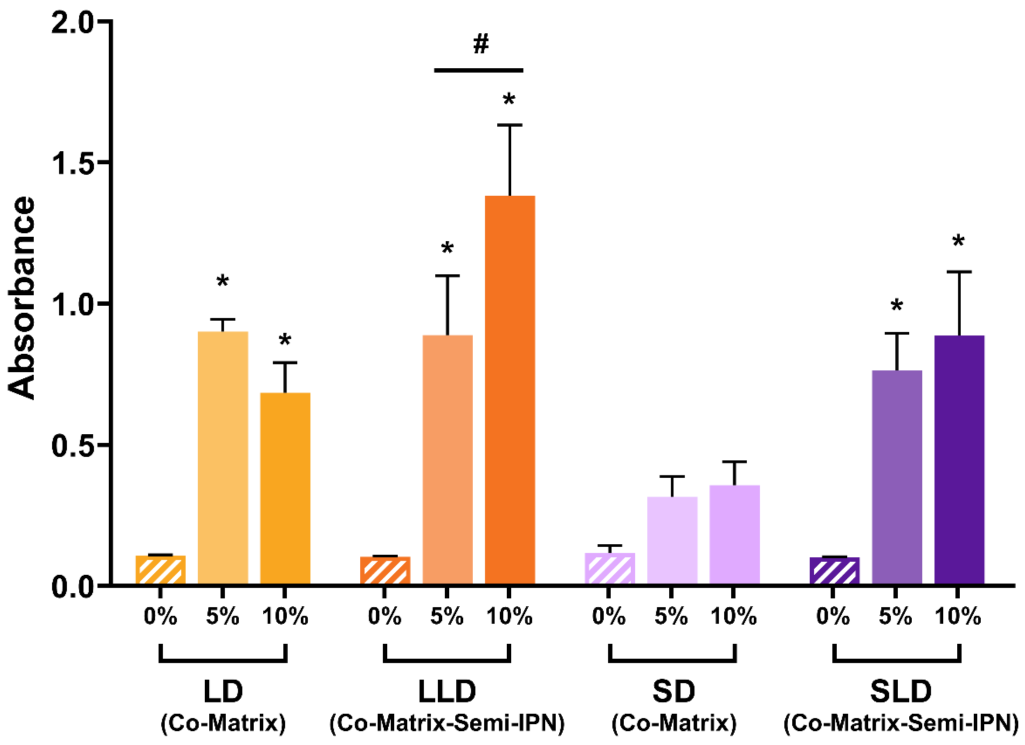

Figure S2. Absorbance of scaffolds stained with Alizarin Red S; *$p < 0.05$ vs. analogous 0% BG scaffold; #$p < 0.05$ versus analogous 5% and 10% BG scaffolds. % refers to wt% of BG.

Table S4. Absorbance values of scaffolds stained with Alizarin Red S. % refers to wt% of BG.

Scaffold	Absorbance
**LD-0%**	0.11 ± 0.00
**LD-5%**	0.90 ± 0.04
**LD-10%**	0.68 ± 0.11
**LLD-0%**	0.10 ±0.00
**LLD-5%**	0.89 ± 0.21
**LLD-10%**	1.38 ±0.25
**SD-0%**	0.12 ± 0.03
**SD-5%**	0.32 ±0.07
**SD-10%**	0.36 ± 0.08
**SLD-0%**	0.10 ± 0.00
**SLD-5%**	0.76 ± 0.13
**SLD-10%**	0.89 ± 0.23

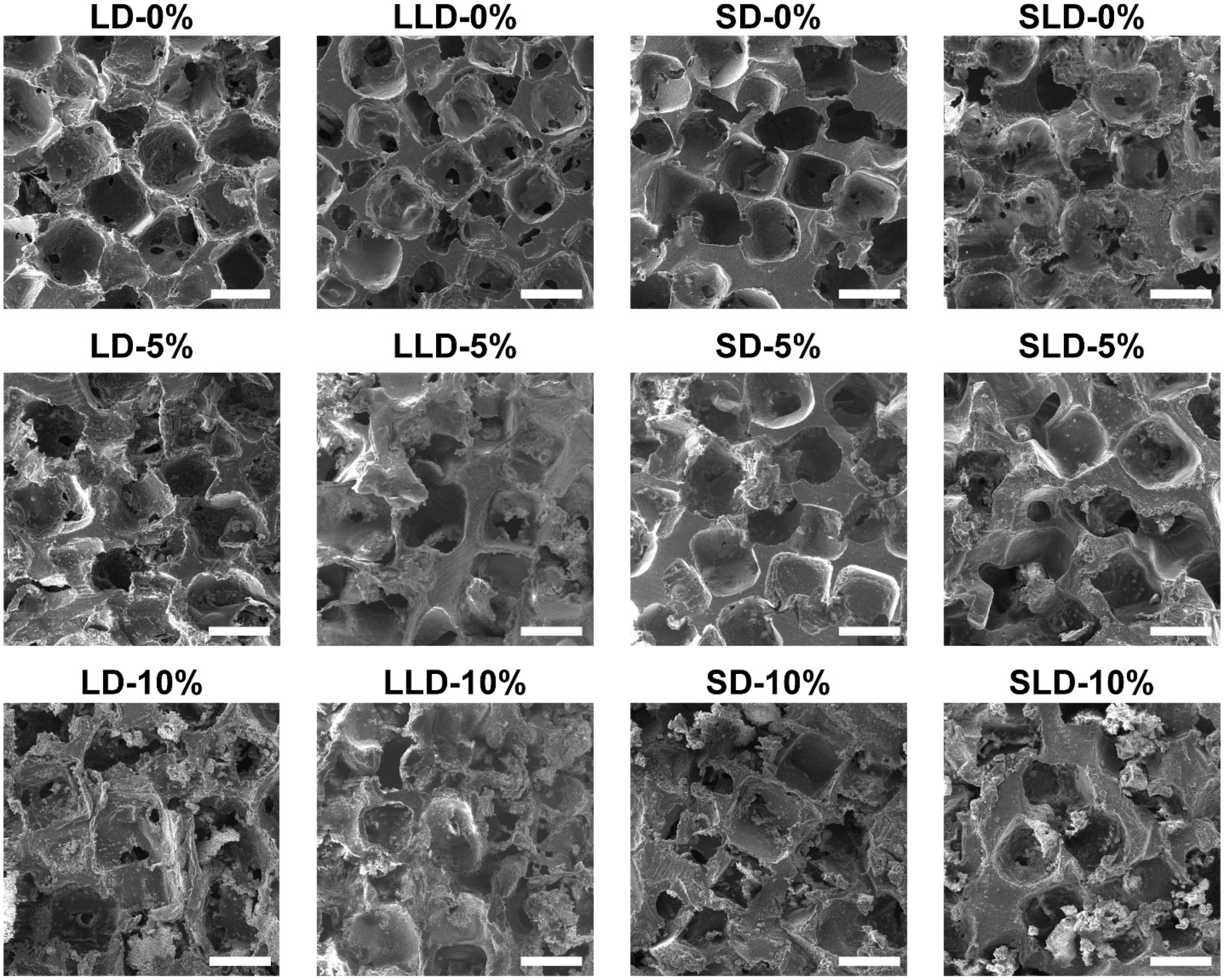

Figure S3. SEM images of scaffolds. (Scale bars = 200 μm). % refers to wt% of BG.

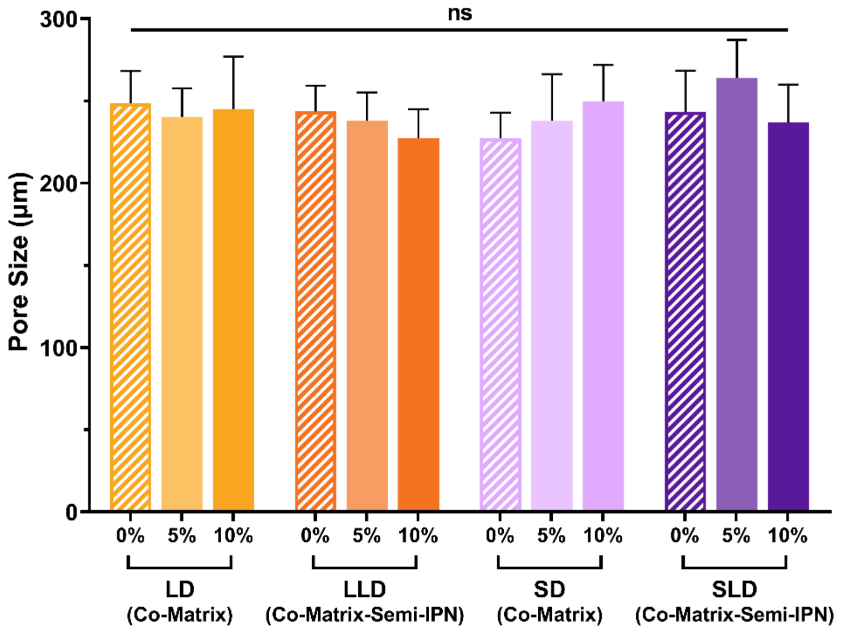

Figure S4. Pore size of scaffolds; *ns* = no significant difference versus analogous 0% BG scaffold. % refers to wt% of BG.

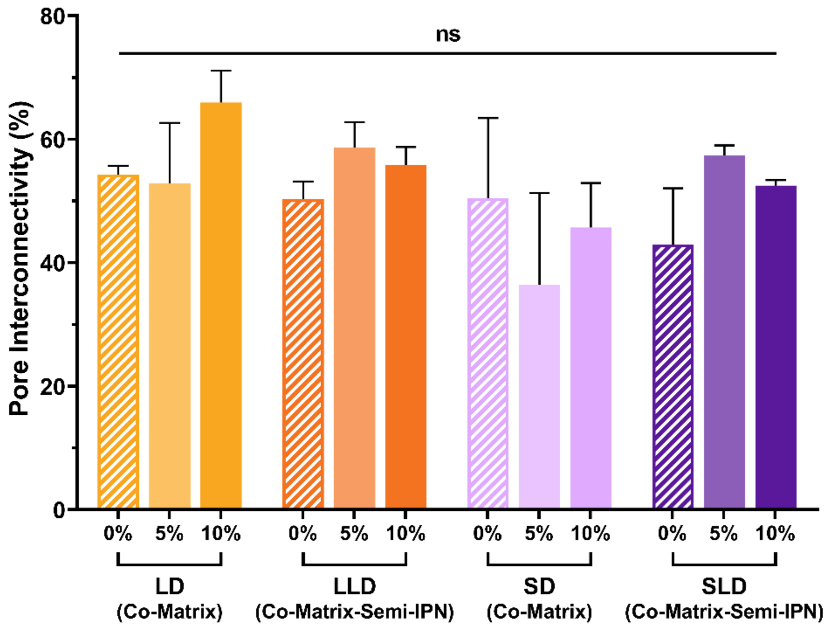

Figure S5. Pore interconnectivity of scaffolds; *ns* = no significant difference versus analogous 0% BG scaffold. % refers to wt% of BG.

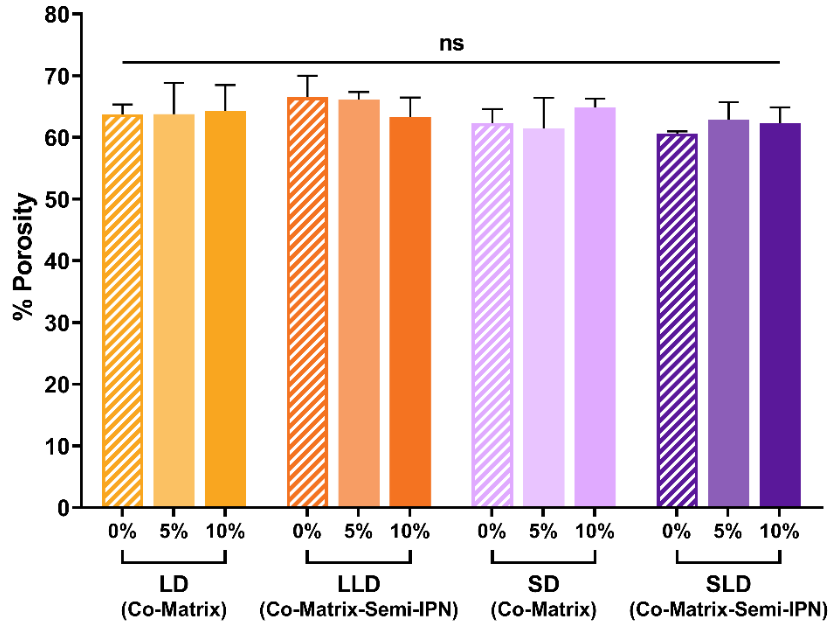

Figure S6. Porosity (%) of scaffolds; *ns* = no significant difference versus analogous 0% BG scaffold. % refers to wt% of BG.

Table S5. Scaffold pore properties. % refers to wt% of BG.

Scaffold	Pore Size (μm)	Pore Interconnectivity (%)	Porosity (%)
**LD-0%**	248 ± 20	54.30 ± 1.41	63.76 ± 1.58
**LD-5%**	240 ± 17	52.84 ± 9.82	63.78 ± 5.06
**LD-10%**	245 ± 32	65.95 ± 5.16	64.27 ± 4.24
**LLD-0%**	244 ± 16	50.31 ± 2.84	66.56 ± 3.43
**LLD-5%**	238 ± 17	58.67 ± 4.10	66.10 ± 1.27
**LLD-10%**	227 ± 18	55.82 ± 2.94	63.31 ± 3.16
**SD-0%**	227 ± 16	50.49 ± 12.95	62.33 ± 2.26
**SD-5%**	238 ± 28	36.42 ± 14.89	61.44 ± 4.97
**SD-10%**	250 ± 22	45.71 ± 7.21	64.82 ± 1.48
**SLD-0%**	243 ± 25	42.98 ± 9.10	60.61 ± 0.38
**SLD-5%**	264 ± 23	57.36 ± 1.67	62.92 ± 2.82
**SLD-10%**	237 ± 23	52.49 ± 0.91	62.34 ± 2.56

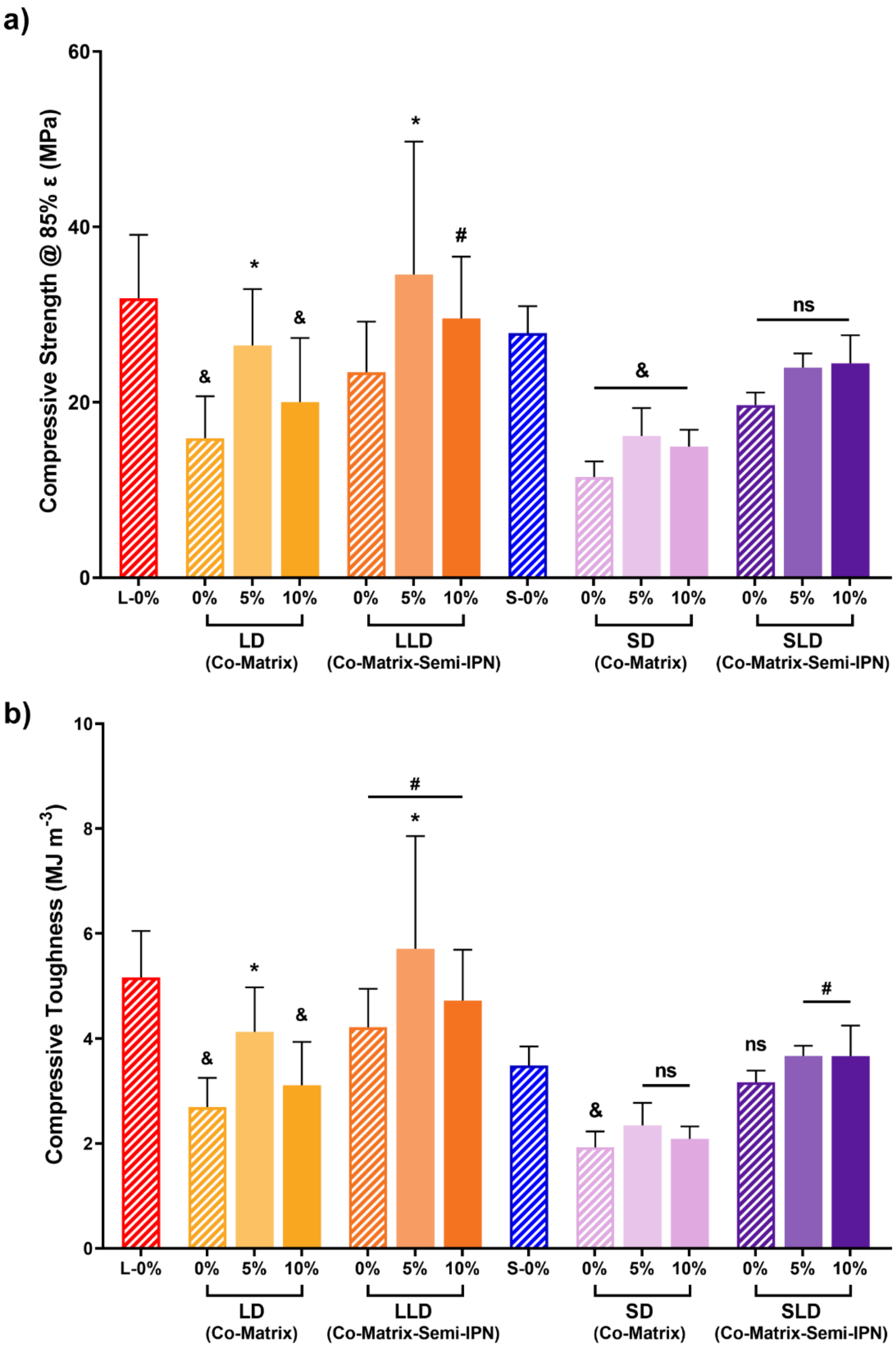

Figure S7. (a) Compressive strength and **(b)** compressive toughness of scaffolds; $^{\&}p < 0.05$ vs. analogous PCL-only scaffold; $^{*}p < 0.05$ vs. 0% BG scaffold of the same macromer solution; $^{\#}p < 0.05$ vs. analogous co-matrix scaffold of similar BG wt%; ns = no significant difference vs. analogous PCL-only scaffold. % refers to wt% of BG. (Note: L-0% and S-0% data previously reported).[1]

Table S6. Compressive mechanical properties of scaffolds. % refers to wt% of BG. (Note: L-0% and S-0% previously data reported).[1]

Scaffold	Modulus (MPa)	Strength (MPa)	Toughness (MJ m^{-3})
**L-0%**	7.38 ± 1.87	31.85 ± 7.23	5.16 ± 0.89
**LD-0%**	5.87 ± 1.00	15.91 ± 4.78	2.69 ± 0.56
**LD-5%**	6.09 ± 1.21	26.47 ± 6.44	4.12 ± 0.85
**LD-10%**	4.48 ± 0.67	20.01 ± 7.32	3.11 ± 0.82
**LLD-0%**	9.15 ± 2.30	23.43 ± 5.76	4.21 ± 0.73
**LLD-5%**	11.34 ± 4.76	34.56 ± 15.17	5.71 ± 2.15
**LLD-10%**	6.54 ± 3.11	29.55 ± 7.05	4.72 ± 0.97
**S-0%**	2.54 ± 0.27	27.88 ± 3.08	3.49 ± 0.36
**SD-0%**	3.18 ± 0.52	11.46 ± 1.79	1.92 ± 0.30
**SD-5%**	3.07 ± 0.70	16.14 ± 3.20	2.34 ± 0.43
**SD-10%**	2.73 ± 0.13	14.96 ± 1.90	2.09 ± 0.23
**SLD-0%**	8.10 ± 1.15	19.69 ± 1.41	3.16 ± 0.22
**SLD-5%**	9.40 ± 1.68	23.95 ± 1.61	3.67 ± 0.19
**SLD-10%**	6.75 ± 1.57	24.42 ± 3.23	3.67 ± 0.58

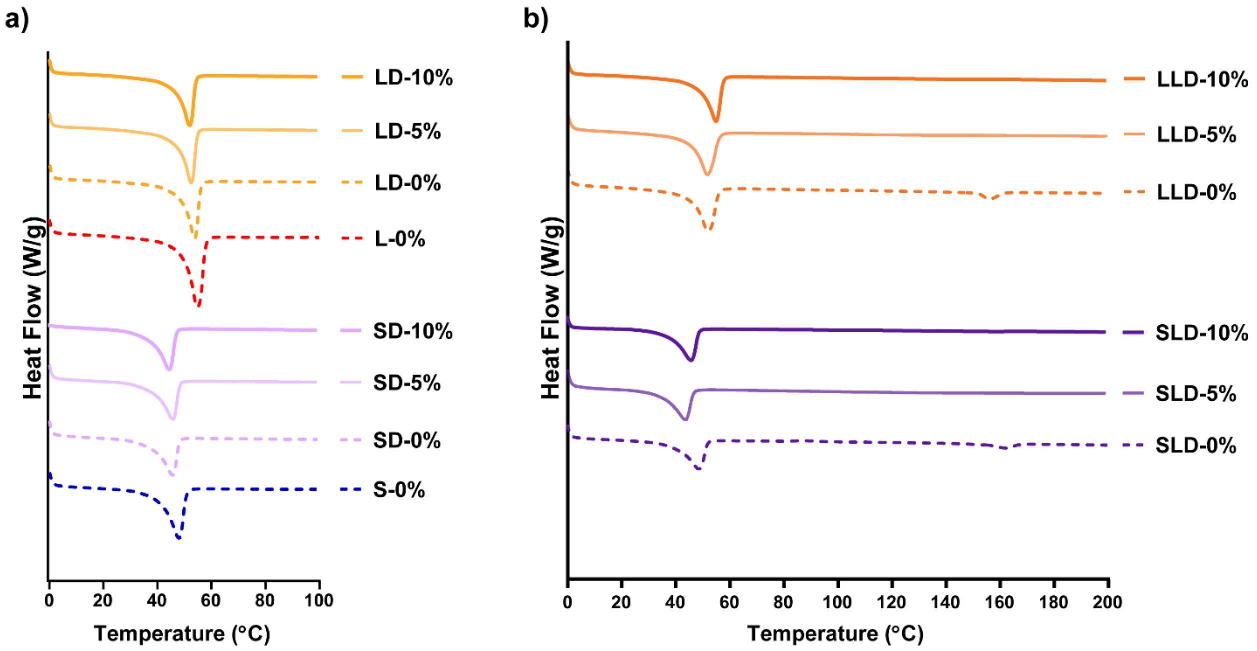

Figure S8. Scaffold thermograms for **(a)** PCL-only and co-matrix compositions and **(b)** co-matrix-semi-IPN compositions. % refers to wt% of BG. (Note: L-0% and S-0% data previously reported).[1]

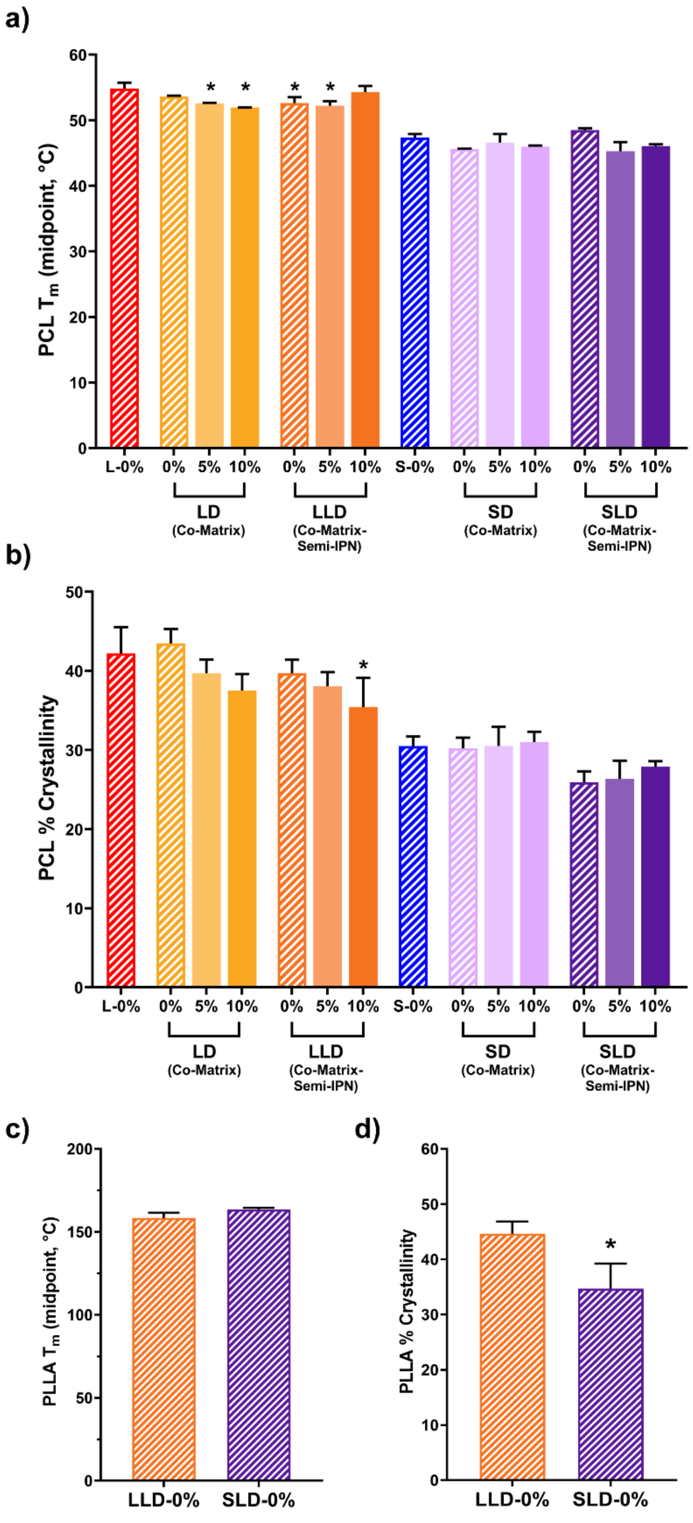

Figure S9. Scaffold thermal properties. **(a)** Midpoint T_m of PCL; *$p < 0.05$ vs. analogous PCL-only scaffold. **(b)** PCL % crystallinity; *$p < 0.05$ vs. analogous PCL-only scaffold. **(c)** Midpoint T_m of PLLA. **(d)** PLLA % crystallinity; $p < 0.05$ vs. LLD-0% scaffold. % refers to wt% of BG. (Note: L-0% and S-0% data previously reported).[1]

Table S7. Thermal properties of scaffolds. % refers to wt% of BG. (Note: L-0% and S-0% data previously reported).[1]

Scaffold	PCL		PLLA	
	T_m midpoint (°C)	% crystallinity	T_m midpoint (°C)	% crystallinity
**L-0%**	54.82 ± 0.91	42.20 ± 3.32	-	-
**LD-0%**	53.62 ± 0.12	43.46 ± 1.82	-	-
**LD-5%**	52.58 ± 0.09	39.70 ± 1.74	-	-
**LD-10%**	51.95 ± 0.01	37.50 ± 2.09	-	-
**LLD-0%**	52.63 ± 0.88	39.71 ± 1.70	158.41 ± 3.17	44.66 ± 2.20
**LLD-5%**	52.22 ± 0.71	38.06 ± 1.79	-	-
**LLD-10%**	54.29 ± 0.94	35.44 ± 3.70	-	-
**S-0%**	47.38 ± 0.53	30.50 ± 1.21	-	-
**SD-0%**	45.62 ± 0.04	30.21 ± 1.34	-	-
**SD-5%**	46.58 ± 1.32	30.52 ± 2.44	-	-
**SD-10%**	45.98 ± 0.16	31.02 ± 1.30	-	-
**SLD-0%**	48.48 ± 0.30	25.93 ± 1.35	163.52 ± 1.04	34.71 ± 4.54
**SLD-5%**	45.30 ± 1.37	26.36 ± 2.29	-	-
**SLD-10%**	46.06 ± 0.28	27.88 ± 0.72	-	-

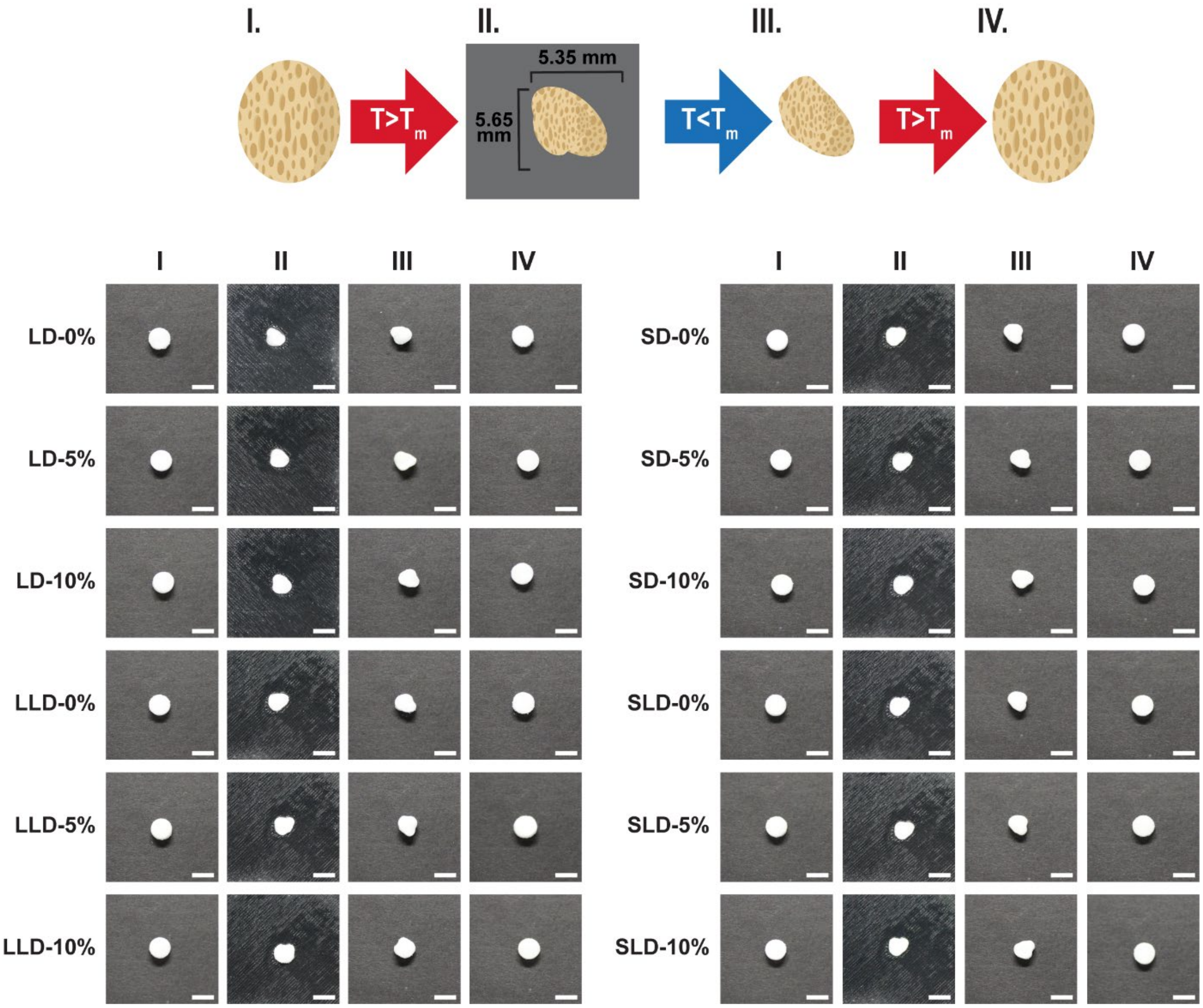

Figure S10. Qualitative shape memory testing of scaffolds. **[I]** Initial scaffold at room temperature. **[II]** Scaffold submerged in warm water 5 °C higher than its PCL's T_m and immediately press-fit into the irregular defect. **[III]** After cooling (3 minutes), the scaffold was removed from model defect. **[IV]** The scaffold was submerged in warm water once again and allowed to shape recover. (Scale bars = 6 mm). % refers to wt% of BG.

Table S8. Quantitative shape memory test results. % refers to wt% of BG.

Scaffold	Shape Fixity (R_f, %)	Shape Recovery (R_r, %)
**LD-0%**	98.90 ± 0.60	104.18 ± 2.09
**LD-5%**	95.31 ± 4.64	99.96 ± 1.27
**LD-10%**	98.33 ± 0.42	98.75 ± 0.81
**LLD-0%**	98.86 ± 0.61	98.82 ± 1.46
**LLD-5%**	99.40 ± 0.35	101.12 ± 0.60
**LLD-10%**	98.86 ± 1.11	101.53 ± 1.97
**SD-0%**	98.86 ± 0.76	101.78 ± 1.07
**SD-5%**	97.86 ± 1.82	99.93 ± 2.65
**SD-10%**	99.33 ± 0.31	96.30 ± 6.84
**SLD-0%**	99.33 ± 0.58	99.26 ± 1.55
**SLD-5%**	99.20 ± 0.20	100.54 ±1.24
**SLD-10%**	99.06 ± 0.31	102.15 ± 1.05

\

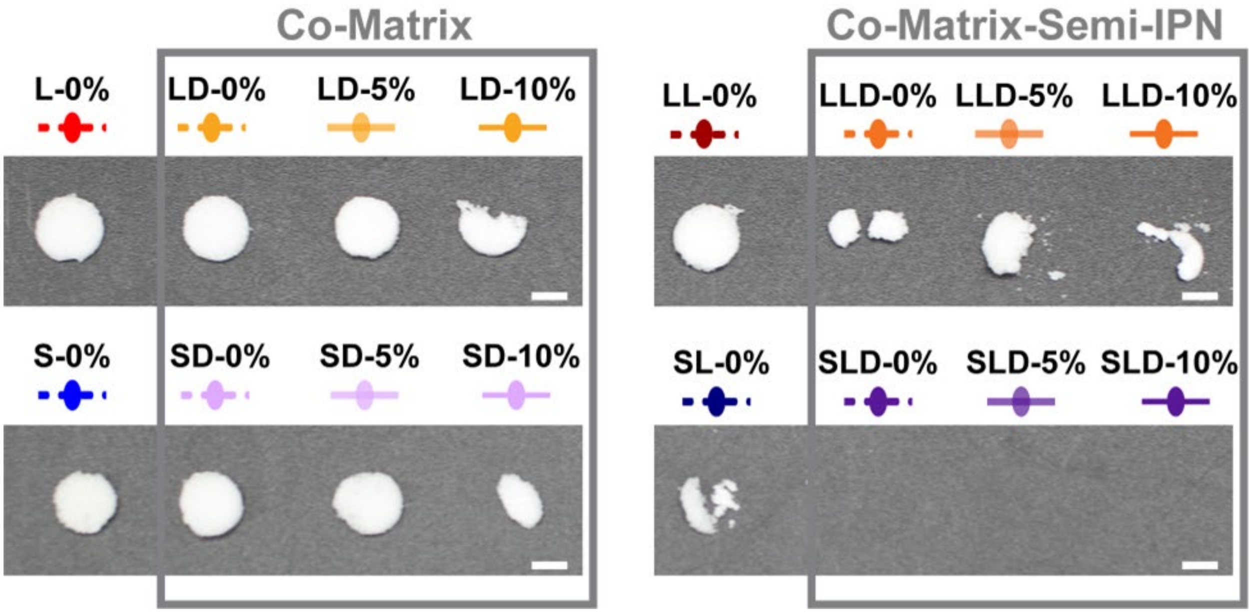

Figure S11. Photo-series of scaffold specimens at t = 5-days (0.2 M NaOH, 37 °C). (Scale bars = 4 mm). % refers to wt% of BG. (Note: L-0%, LL-0%, S-0%, and SL-0% data previously reported).[1]

Table S9. Mass remaining (%) of scaffolds after accelerated degradation testing. % refers to wt% of BG. (Note: L-0%, LL-0%, S-0%, and SL-0% data previously reported).[1]

Scaffold	24 hr	48 hr	72 hr	96 hr	120 hr	144 hr	168 hr
**L-0%**	96.01 ± 1.69	96.08 ± 2.18	94.19 ± 1.20	93.02 ± 1.13	89.49 ± 3.79	83.80 ± 8.56	64.48 ± 6.64
**LD-0%**	95.55 ± 3.58	96.51 ± 1.49	90.31 ± 2.31	88.01 ± 1.11	82.03 ± 2.34	76.07 ± 6.48	69.28 ± 3.92
**LD-5%**	96.77 ± 0.93	92.01 ± 2.63	83.86 ± 4.75	80.07 ± 0.39	73.30 ± 0.84	55.91 ± 5.85	48.44 ± 3.88
**LD-10%**	94.73 ± 1.28	93.67 ± 1.83	79.34 ± 4.58	71.19 ± 11.50	65.27 ± 9.19	50.67 ± 7.36	52.11 ± 5.13
**LL-0%**	86.81 ± 0.21	79.89 ± 2.45	76.33 ± 1.47	68.02 ± 0.17	60.91 ± 1.85	50.45 ± 1.64	33.91 ± 7.93
**LLD-0%**	89.81 ± 2.29	83.29 ± 7.56	78.59 ± 5.23	63.08 ± 11.62	44.25 ± 4.90	25.95 ± 5.09	17.44 ± 6.10
**LLD-5%**	90.25 ± 1.71	83.33 ± 2.66	71.48 ± 10.30	38.29 ± 34.04	54.49 ± 11.23	42.02 ± 3.72	31.27 ± 2.79
**LLD-10%**	70.46 ± 6.46	51.74 ± 2.66	41.72 ± 4.98	26.72 ± 8.48	26.08 ± 5.24	18.26 ± 3.02	2.18 ± 3.78
**S-0%**	97.24 ± 0.69	94.41 ± 0.93	83.05 ± 7.73	72.55 ± 9.50	59.52 ± 15.35	46.71 ± 14.90	7.82 ± 8.94
**SD-0%**	96.92 ± 1.47	97.54 ± 2.22	87.48 ± 2.70	80.71 ± 6.77	73.81 ± 7.70	63.46 ± 11.60	49.06 ± 24.56
**SD-5%**	96.58 ± 1.04	92.61 ± 1.00	90.11 ± 2.17	76.28 ± 20.71	69.38 ± 9.25	58.17 ± 3.00	38.64 ± 8.32
**SD-10%**	92.67 ± 2.33	86.89 ± 2.03	68.09 ± 5.17	53.92 ± 6.85	36.69 ± 11.59	35.69 ± 0.88	16.67 ± 6.35
**SL-0%**	85.95 ± 3.49	68.95 ± 1.83	53.30 ± 4.91	22.88 ± 8.31	17.08 ± 9.09	4.32 ± 7.49	0.00 ± 0.00
**SLD-0%**	81.69 ± 2.20	56.42 ± 7.27	39.96 ± 6.40	23.57 ± 40.83	0.00 ± 0.00	0.00 ± 0.00	0.00 ± 0.00
**SLD-5%**	87.85 ± 1.42	61.73 ± 15.04	47.91 ± 4.14	31.03 ± 5.27	0.00 ± 0.00	0.00 ± 0.00	0.00 ± 0.00
**SLD-10%**	86.46 ± 13.12	59.37 ± 17.25	43.39 ± 9.91	4.78 ± 8.29	0.00 ± 0.00	0.00 ± 0.00	0.00 ± 0.00

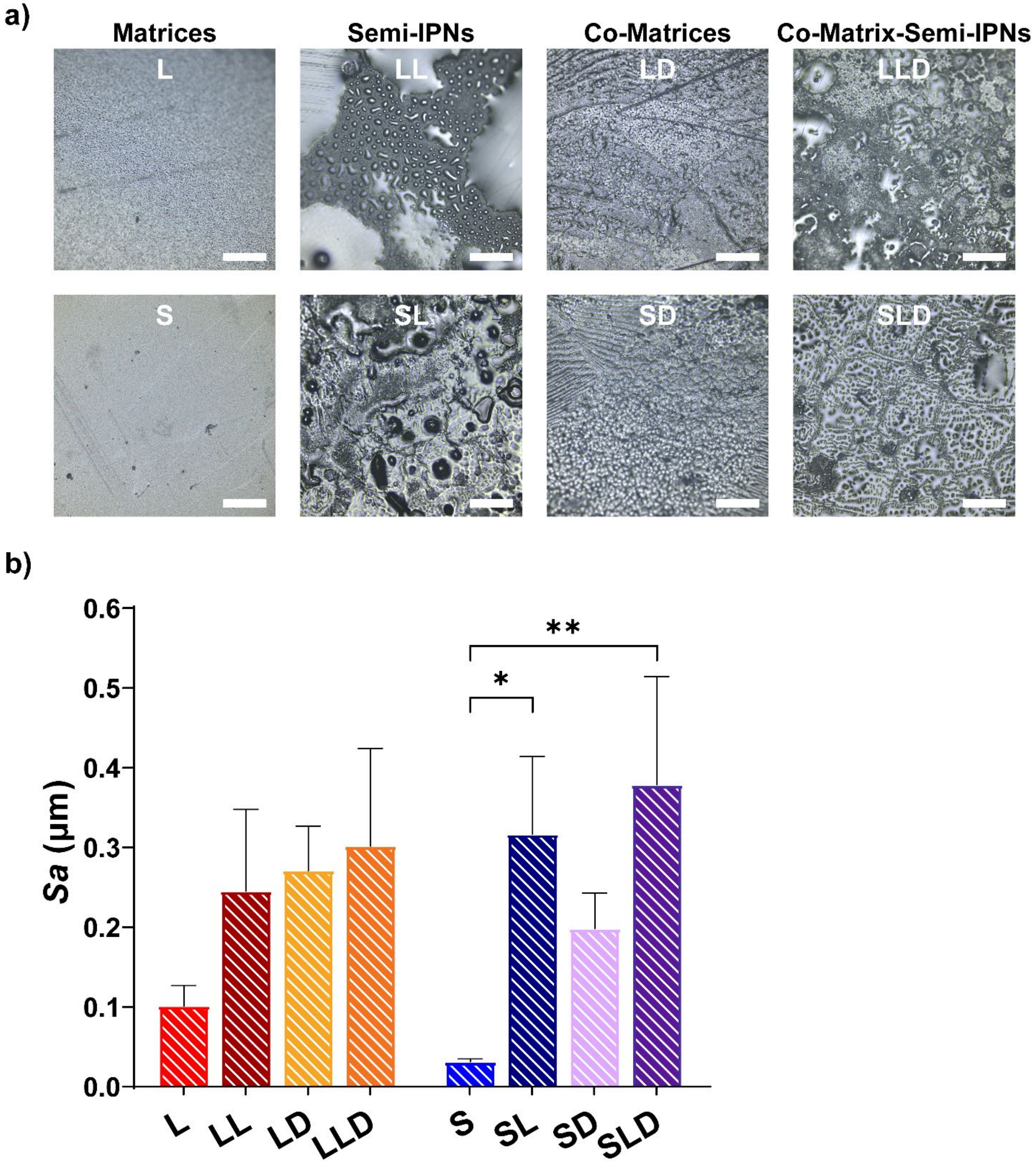

Figure S12. (a) Scanning confocal microscopy images of films. (Scale bars = 250 µm). **(b)** Surface roughness of films; *$p < 0.05$; **$p < 0.01$ vs. analogous PCL-only film.

Table S10. Area roughness (arithmetical mean height, *Sa*) of films.

Film	*Sa* (µm)
**L**	0.10 ± 0.03
**LL**	0.25 ± 0.10
**LD**	0.27 ± 0.06
**LLD**	0.30 ± 0.12
**S**	0.03 ± 0.00
**SL**	0.32 ± 0.10
**SD**	0.20 ± 0.05
**SLD**	0.38 ± 0.14

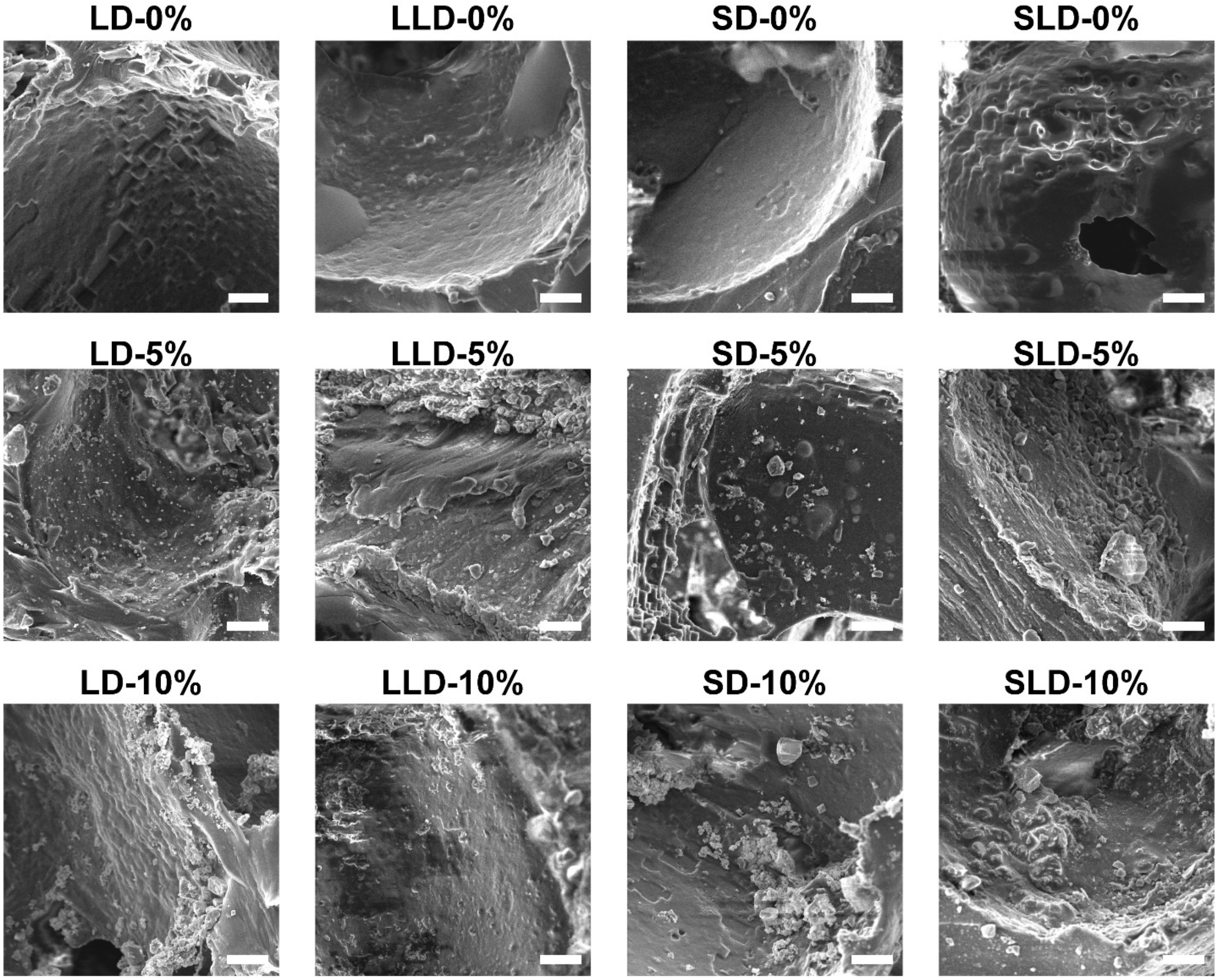

Figure S13. SEM images of scaffolds prior to 1X SBF exposure. (Scale bars = 20 μm). % refers to wt% of BG.

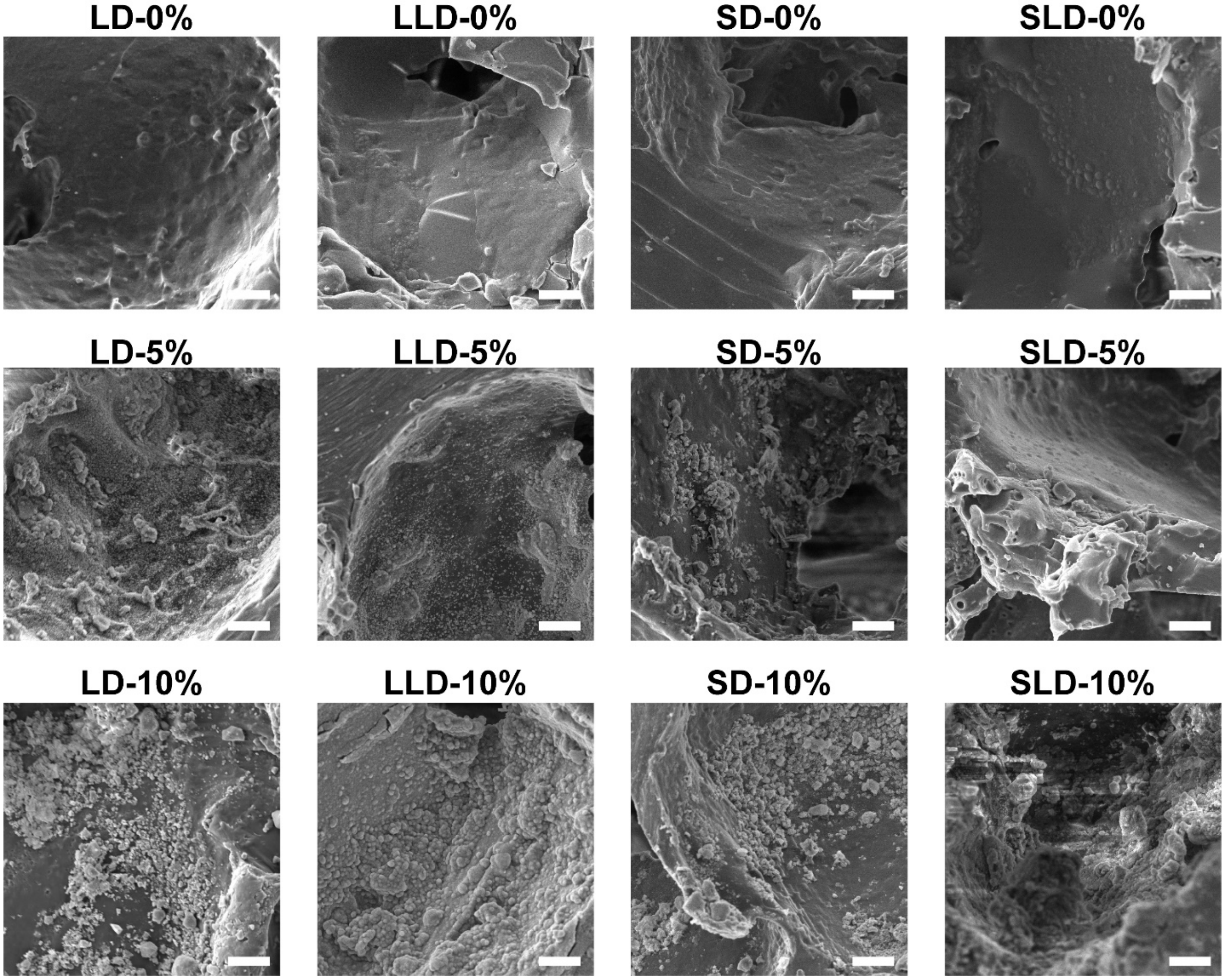

Figure S14. SEM images of scaffolds after 1 day of exposure to 1X SBF. (Scale bars = 20 μm). % refers to wt% of BG.

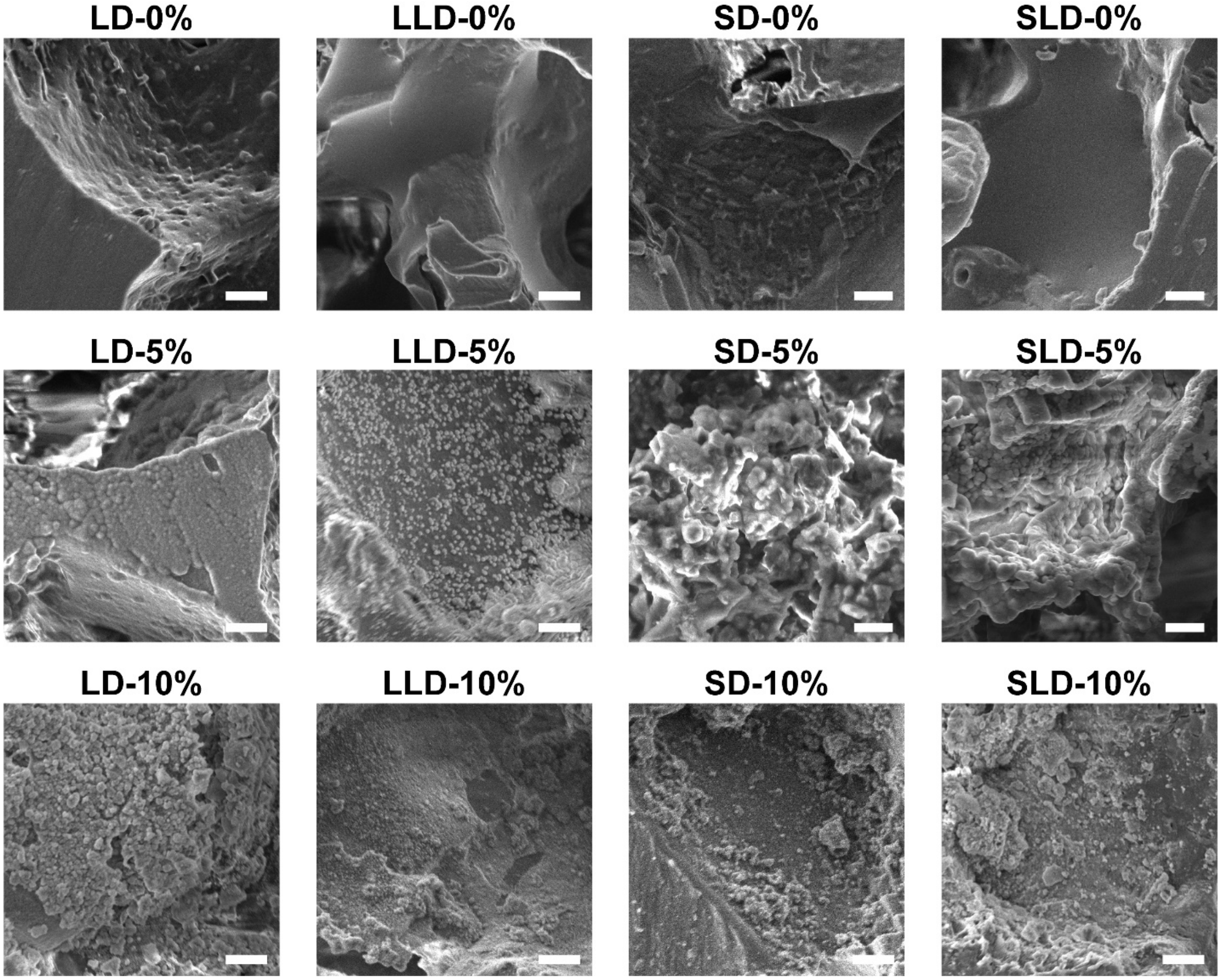

Figure S15. SEM images of scaffolds after 2 weeks of exposure to 1X SBF. (Scale bars = 20 μm). % refers to wt% of BG.

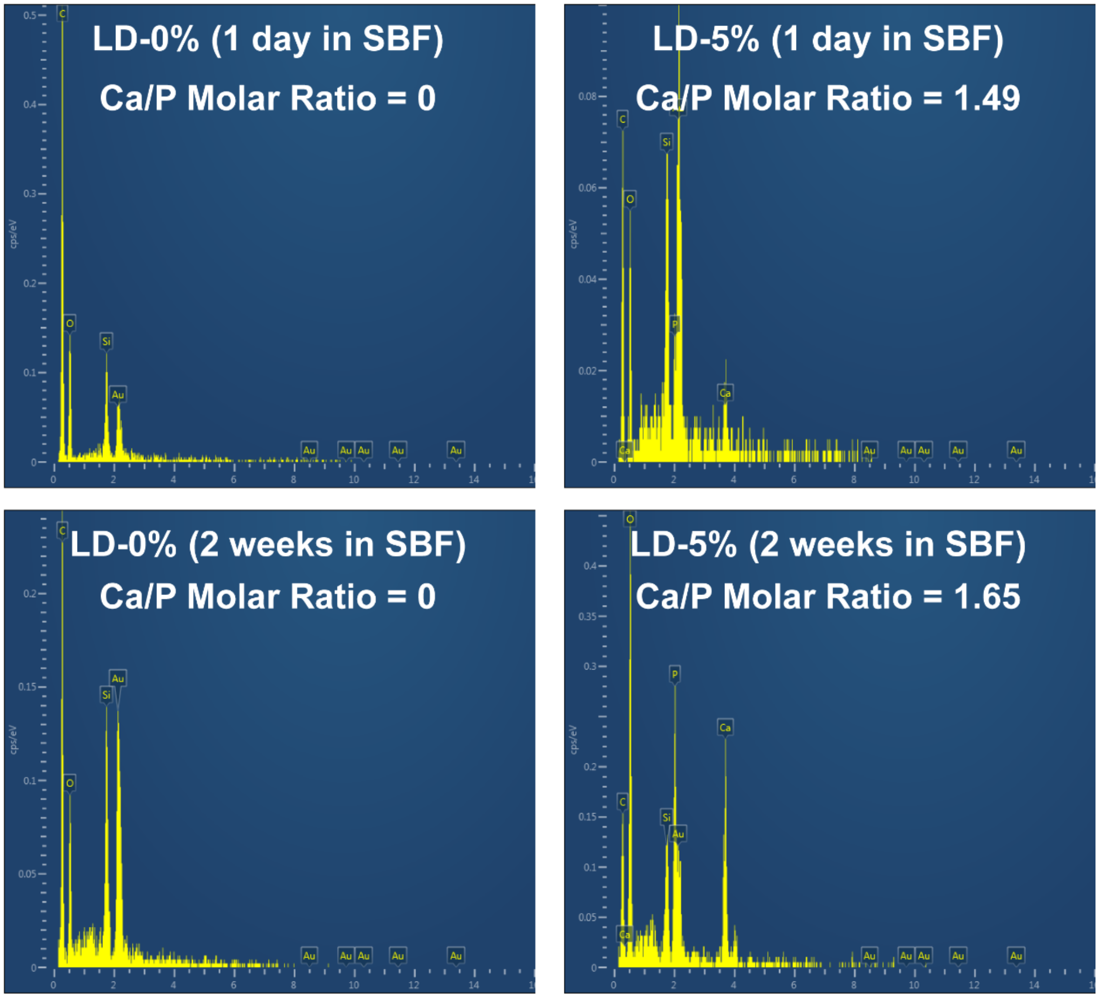

Figure S16. Representative EDS spectra of PCL/PDMS co-matrix scaffolds with no BG (**LD-0%**) and with 5 wt% BG (**LD-5%**) after 1 day and 2 weeks of 1X SBF exposure. % refers to wt% of BG.

References

[1] B.M. Nitschke, E.A. Butchko, M.N. Wahby, K.M. Breining, A.E. Konz, M.A. Grunlan, Shape Memory Polymer Bioglass Composite Scaffolds Designed to Heal Complex Bone Defects, ACS Biomater. Sci. Eng. 10 (2024) 6509-6519, https://doi.org/10.1021/acsbiomaterials.4c01073.
